# Systematics of Neotropical microteiid lizards (Gymnophthalmidae, Cercosaurinae), with the description of a new genus and species from the Andean montane forests

**DOI:** 10.3897/zookeys.774.25332

**Published:** 2018-07-16

**Authors:** Jiří Moravec, Jiří Šmíd, Jan Štundl, Edgar Lehr

**Affiliations:** 1 Department of Zoology, National Museum, Cirkusová 1740, 193 00 Prague 9, Czech Republic; 2 Department of Zoology, Faculty of Science, Charles University in Prague, Viničná 7, Prague, Czech Republic; 3 Department of Biology, Illinois Wesleyan University, P.O. Box 2900, IL 61701, USA

**Keywords:** Andes, arboreality, phylogeny, reptile diversity, *Selvasaura* gen. n., *Selvasaura brava* sp. n., taxonomy

## Abstract

Cercosaurine lizards (subfamily Cercosaurinae of the family Gymnophthalmidae) represent a substantial component of the reptile fauna in the Neotropics. Several attempts have been made to reconstruct the phylogenetic relationships within this group, but most studies focused on particular genera or regions and did not cover the subfamily as a whole. In this study, material from the montane forests of Peru was newly sequenced. In combination with all cercosaurine sequences available on GenBank, an updated phylogeny of Cercosaurinae is provided. Monophyly was not supported for three of the currently recognised genera (*Echinosaura*, *Oreosaurus*, and *Proctoporus*). The genus *Proctoporus* is formed by five monophyletic groups, which should be used in future taxonomic revisions as feasible entities. Recognition of two previously identified undescribed clades (Unnamed clades 2 and 3) was supported and yet another undescribed clade (termed here Unnamed clade 4), which deserves recognition as an independent genus, was identified herein. *Selvasaura brava*, a new genus and new species of arboreal gymnophthalmid lizard is described from the montane forests of the Pui Pui Protected Forest, Provincia de Chanchamayo, Región Junín, Peru. The new species is characterised by its small size (SVL 42.1–45.9 mm), slender body, smooth head shields, presence of paired prefrontal shields, fused anteriormost supraocular and anteriormost superciliary shields, transparent not divided lower palpebral disc, slightly rugose subimbricate rectangular dorsal scales in adults (slightly keeled in juveniles), distinctly smaller but non-granular lateral scales, smooth squared to rectangular ventral scales, and hemipenial lobes large, distinct from the hemipenial body. Phylogenetic affinities of the new genus to the other cercosaurine genera, as well as basal phylogenetic relationships between the other cercosaurine genera remain unresolved.

## Introduction

Gymnophthalmid lizards (family Gymnophthalmidae) represent a substantial component of the reptile fauna in the Neotropics. They are traditionally divided into subfamilies (sometimes referred to as tribes; Pellegrino et al. 2001a; Goicoechea et al. 2016), of which the cercosaurines (Cercosaurinae), with approximately 140 species, form the most species-rich clade. This subfamily is distributed throughout South America and the Andes represent the main centre of its diversity.

Phylogenetic analyses of cercosaurines based on genetic data started appearing after 2000 (Pellegrino et al. 2001a) and have since progressed considerably with respect to taxon sampling. Recent phylogenetic studies have brought new findings that resulted in taxonomic changes at the level of genera: some genera were synonymised, others resurrected, and new genera described. For example, Doan and Castoe (2005) proposed the new generic names *Potamites*, *Petracola*, and *Riama* for some species formerly ranked under *Neusticurus* and *Proctoporus*. Subsequently, Torres-Carvajal et al. (2016) described *Gelanesaurus* for some species that had formerly belonged to *Potamites*, after they found the latter to be paraphyletic. Most recently, Sánchez-Pacheco et al. (2017b) described *Andinosaura* and resurrected *Oreosaurus* to accommodate the polyphyly of *Riama*. At the moment, Cercosaurinae consists of 15 formally described genera: *Anadia*, *Andinosaura*, *Cercosaura*, *Echinosaura*, *Euspondylus*, *Gelanesaurus*, *Macropholidus*, *Neusticurus*, *Oreosaurus*, *Petracola*, *Pholidobolus*, *Placosoma*, *Potamites*, *Proctoporus*, and *Riama*. Recently, Torres-Carvajal et al. (2016) identified three more evolutionary lineages within cercosaurines at the level of genera, some of which are still awaiting formal descriptions.

Despite the undeniable advances in untangling the cercosaurine tree, there are still genera and species for which monophyly has failed to be proven (Torres-Carvajal et al. 2016). Moreover, basically every new phylogenetic study published to date brought evidence for cryptic species being present (Goicoechea et al. 2012; Torres-Carvajal et al. 2015, 2016; Sánchez-Pacheco et al. 2017b). The amount of overall cryptic diversity within all cercosaurines is not straightforward to gauge because most previous phylogenies had a narrow focus on particular genera (Doan et al. 2005; Goicoechea et al. 2012; Torres-Carvajal and Mafla-Endara 2013; Aguirre-Peñafiel et al. 2014) or regions (Kok et al. 2012), or had species represented by a single sample (Pellegrino et al. 2001b; Kok 2015). The most comprehensive phylogenetic reconstructions of higher clades were published recently by Torres-Carvajal et al. (2015, 2016), Goicoechea et al. (2016), and Sánchez-Pacheco et al. (2017b). The aim of this study was to contribute to the phylogeny of Cercosaurinae by inferring the phylogenetic placement of new material collected during recently conducted surveys with a special emphasis on the Pui Pui Protected Forest in Peru and its surroundings.

The Pui Pui Protected Forest (Bosque de Protección Pui Pui, hereafter PPPF) is located in the Selva Central of Peru and covers 60,000 hectares (30% montane forest, 70% puna habitats) between 1700 and 4500 m a.s.l. (SERNANP 2010). We surveyed the herpetofauna of the PPPF in upper montane forests and high Andean grasslands (puna) in 2012, 2013, and 2014 in order to record the amphibian and reptile species richness and to evaluate their conservation status. We have discovered several new species of amphibians and reptiles, e.g., frogs of the genera *Pristimantis*, and *Phrynopus*, and lizards of the genera *Euspondylus, Potamites*, and *Proctoporus* (Lehr and Moravec 2017; Lehr et al. 2017a, b; work in progress), which suggests that biodiversity of this region is still far from being fully inventoried. Additionally, the material collected in PPPF contained a new gymnophthalmid lizard that was morphologically difficult to assign to the currently recognised genera.

## Materials and methods

### Material for phylogenetic analyses

We assembled a genetic dataset that included sequences for the subfamily Cercosaurinae available on GenBank. Additionally, we newly sequenced 38 samples of nine genera (*Anadia*, *Cercosaura*, *Euspondylus*, *Pholidobolus*, *Potamites*, *Proctoporus*, the new genus described herein and two unnamed genera; Table 1, Suppl. material 1: Fig. S1) deposited in the SMF, NMP, IWU, JCM, and MUSM collections (for collection acronyms see below). All genera that are presently recognised to form the content of the subfamily (Sánchez-Pacheco et al. 2017b) were included in the dataset, represented as follows: *Anadia* (3 of 18 described species included), *Andinosaura* (9 of 11 described species included), *Cercosaura* (10 of 14 described species included), *Echinosaura* (5 of 7 described species included), *Euspondylus* (1 of 11 described species included), *Gelanesaurus* (2 of 2 described species included), *Macropholidus* (3 of 4 described and one yet undescribed species included), *Neusticurus* (2 of 5 described species included), *Oreosaurus* (4 of 6 described and one yet undescribed species included), *Petracola* (2 of 4 described species included), *Pholidobolus* (8 of 9 described and one yet undescribed species included), *Placosoma* (2 of 4 described species included), *Potamites* (6 of 7 described species included), *Proctoporus* (15 of 17 described and eight yet undescribed species included), *Riama* (14 of 15 described and three yet undescribed species included). We also included the unnamed clades identified by Torres-Carvajal et al. (2016) that could not be assigned to any described genus.

**Table 1.** List of material newly sequenced for this study. Sample codes are those shown in tree figures. Locality numbers refer to those in Fig. 1. Upper indexes H and P denote holotype and paratypes, respectively.

Species	Sample code	Voucher code	GenBank Accession				Locality (Locality no.)	Lat	Long
			12S	16S	cytb	cmos			
*Anadia ocellata*	SMF90095	SMF 90095	MH579588	MH579625	MH579659	-	Panama, Chiriqui, Santa Clara (1)	8.833, -82.782	
*Cercosaura argulus*	184	NMP6V 72184	MH579589	MH579626	MH579660	MH579686	Bolivia, Pando, Nacebe (2)	-11.000, -67.417	
*Cercosaura eigenmanni*	609	NMP6V 72609	MH579590	MH579627	-	-	Bolivia, Pando, Bioceanica (3)	-11.133, -69.367	
*Cercosaura eigenmanni*	112	NMP6V 73112	MH579591	MH579628	MH579661	-	Bolivia, Pando, Canada (4)	-11.750, -67.133	
*Cercosaura oshaughnessyi*	155/2	NMP6V 71155/2	MH579592	-	-	-	Peru, Loreto, 21 km W of Iquitos (5)	-3.787, -73.429	
*Cercosaura oshaughnessyi*	160/1	NMP6V 71160/1	MH579593	MH579629	MH579662	MH579687	Peru, Loreto, Puerto Almendras (6)	-3.829, -73.376	
*Euspondylus excelsum*	IWU234	IWU 234	MH579594	MH579630	-	MH579688	Peru, Junín, B.P. Pui Pui surr. (7)	-11.096, -75.228	
*Pholidobolus* sp.1	JCM238	JCM 238	MH579595	MH579631	MH579663	MH579689	Peru, Cajamarca, S.N. Tabaconas (8)	-5.157, -79.273	
*Pholidobolus* sp.1	JCM239	JCM 239	MH579596	MH579632	MH579664	MH579690	Peru, Cajamarca, S.N. Tabaconas (8)	-5.157, -79.273	
*Pholidobolus* sp.1	843	MUSM 31843	MH579597	MH579633	MH579665	MH579691	Peru, Cajamarca, S.N. Tabaconas (8)	-5.157, -79.273	
*Pholidobolus ulisesi*	JCM310	JCM 310	MH579598	MH579634	MH579666	MH579692	Peru, Cajamarca, S.N. Tabaconas (8)	-5.157, -79.273	
*Potamites ecpleopus*	186/1	NMP6V 73186/1	MH579599	MH579635	-	-	Peru, Loreto, Anguilla (9)	-3.913, -73.661	
*Potamites ecpleopus*	186/2	NMP6V 73186/2	MH579600	MH579636	-	-	Peru, Loreto, Anguilla (9)	-3.913, -73.661	
*Proctoporus chasqui*	IWU24	MUSM 31108	MH579601	MH579637	MH579667	MH579693	Peru, Pasco, N.P. Yanachaga Chemillen (10)	-10.395, -75.482	
*Proctoporus chasqui*	IWU25	MUSM 31109	MH579602	MH579638	MH579668	MH579694	Peru, Pasco, N.P. Yanachaga Chemillen (10)	-10.395, -75.482	
*Proctoporus chasqui*	IWU50	MUSM 31123	MH579603	MH579639	MH579669	MH579695	Peru, Pasco, N.P. Yanachaga Chemillen (10)	-10.395, -75.482	
*Proctoporus chasqui*	IWU82	MUSM 31142	MH579604	MH579640	MH579670	MH579696	Peru, Pasco, N.P. Yanachaga Chemillen (10)	-10.395, -75.482	
*Proctoporus chasqui*	IWU133	MUSM 31172	MH579605	MH579641	MH579671	MH579697	Peru, Junín, B.P. Pui Pui (11)	-11.255, -74.892	
*Proctoporus spinalis*	IWU119	MUSM 31162	MH579607	MH579643	-	-	Peru, Junín, B.P. Pui Pui surr. (12)	-11.712, -75.089	
*Proctoporus spinalis*	IWU120	IWU 120	MH579608	MH579644	-	MH579699	Peru, Junín, B.P. Pui Pui surr. (12)	-11.712, -75.089	
*Proctoporus* sp.4	IWU358	MUSM 32727	MH579606	MH579642	MH579672	MH579698	Peru, Junín, B.P. Pui Pui, Rio Bravo (13)	-11.211, -74.958	
*Selvasaura brava* gen. et. sp. n.	IWU339	MUSM 32718 ^P^	MH579609	MH579645	MH579673	MH579700	Peru, Junín, B.P. Pui Pui, Rio Bravo (14)	-11.208, -74.955	
*Selvasaura brava* gen. et. sp. n.	IWU340	NMP6V 75655 ^P^	MH579610	MH579646	MH579674	MH579701	Peru, Junín, B.P. Pui Pui, Rio Bravo (14)	-11.208, -74.955	
*Selvasaura brava* gen. et. sp. n.	IWU380	NMP6V 75653 ^P^	MH579611	MH579647	MH579675	MH579702	Peru, Junín, B.P. Pui Pui, Rio Bravo (13)	-11.211, -74.958	
*Selvasaura brava* gen. et. sp. n.	IWU381	MUSM 32738 ^H^	MH579612	MH579648	MH579676	MH579703	Peru, Junín, B.P. Pui Pui, Rio Bravo (13)	-11.211, -74.958	
*Selvasaura brava* gen. et. sp. n.	IWU382	NMP6V 75654 ^P^	MH579613	MH579649	MH579677	MH579704	Peru, Junín, B.P. Pui Pui, Rio Bravo (13)	-11.211, -74.958	
Unnamed clade 2	IWU57	MUSM 31127	-	MH579650	MH579678	MH579705	Peru, Pasco, Bosque de Shollet (15)	-10.676, -75.322	
Unnamed clade 2	IWU114	MUSM 31160	MH579614	-	MH579679	MH579706	Peru, Junín, B.P. Pui Pui surr. (16)	-11.665, -75.037	
Unnamed clade 2	IWU165	MUSM 31188	MH579615	MH579651	MH579680	MH579707	Peru, Junín, B.P. Pui Pui, Tarhuish (17)	-11.378, -74.937	
Unnamed clade 2	IWU287	MUSM 32973	MH579616	-	-	MH579708	Peru, Junín, Toldopampa (18)	-11.484, -74.891	
Unnamed clade 2	IWU288	NMP6V 75084	MH579617	MH579652	MH579681	MH579709	Peru, Junín, Toldopampa (18)	-11.484, -74.891	
Unnamed clade 2	IWU296	MUSM 31978	MH579618	MH579653	MH579682	-	Peru, Junín, B.P. Pui Pui, Hatunpata (19)	-11.302, -75.026	
Unnamed clade 2	IWU320	MUSM 31991	MH579619	MH579654	MH579683	MH579710	Peru, Junín, B.P. Pui Pui, Trancapampa (20)	-11.297, -75.013	
Unnamed clade 2	IWU325	MUSM 31994	MH579620	MH579655	MH579684	MH579711	Peru, Junín, B.P. Pui Pui, Antuyo Bajo (21)	-11.315, -74.993	
Unnamed clade 2	90	NMP6V 75090	MH579621	MH579656	-	-	Peru, Junín, Maraynioc (22)	-11.346, -75.445	
Unnamed clade 2	91	NMP6V 75091	MH579622	MH579657	-	-	Peru, Junín, Maraynioc (22)	-11.346, -75.445	
Unnamed clade 4	EL409	MUSM 27610	MH579623	-	-	-	Peru, Cusco, Alfamayo (23)	-13.066, -72.416	
Unnamed clade 4	ML1352	MUSM 25345	MH579624	MH579658	MH579685	-	Peru, Pasco, N.P. Yanachaga Chemillen surr. (24)	-10.658, -75.298	

To avoid confusion, sequences of all loci were matched with the sample or museum code of the specimen to which they belonged as was used in the original reference (when available). As Cercosaurinae still contains non-monophyletic taxa (see below), we avoided combining sequences of more individuals into chimeric samples, even if they putatively belonged to the same species. As a result, each terminal in the tree represents an existing voucher specimen or tissue sample (Suppl. material 1: Table S1). For those species whose monophyly has been previously confirmed, we included only one (or a few) samples (e.g., *Cercosaura ocellata*; Sturaro et al. 2017).

### DNA extractions, amplifications, and sequencing

Genomic DNA was extracted from ethanol-preserved tissue samples using a Geneaid kit. We PCR-amplified up to four loci, three from the mitochondrial DNA (mtDNA): 12S rRNA (12S), 16S rRNA (16S), cytochrome b (cytb), and the oocyte maturation factor MOS (cmos) from the nuclear DNA. Sanger sequencing of both the forward and reverse strands was carried out at Macrogen (Amsterdam, The Netherlands) using the same primers as for the PCRs. Details on the primers and amplification conditions are given in Table 2. Newly produced sequences were edited and contigs assembled in Geneious v.6 (Kearse et al. 2012). MAFFT v.7 (Katoh and Standley 2013) was used to align all loci individually with the ‘auto’ option selected for all. The Q-INS-I option that considers the secondary structure of RNA and that would therefore have been more suitable for the 12S and 16S datasets could not be used as the number of sequences in both datasets (332, respectively 343) exceeded the allowed limit. The alignments of cytb and cmos were translated into amino acids and no stop codons were detected, suggesting we did not amplify pseudogenes. We applied Gblocks (Castresana 2000) to the 12S and 16S alignments to trim regions that aligned ambiguously. We trimmed the tRNA-end of the ND4 and used only its coding part for the analyses. The final concatenated dataset for the phylogenetic analyses consisted of 2217 bp composed of the following loci with lengths given in parentheses: 12S (325 bp), 16S (454 bp), cytb (307 bp), ND4 (694 bp), and cmos (437 bp).

**Table 2.** Primers and PCR conditions used in this study. Amplicon length refers to the length of the fragment amplified. PCR cycle shows temperatures and times of steps in the cycle itself and not the initial denaturation (94 °C for 5 min) and final elongation (72 °C for 5–10 min) steps.

Gene	Primer	Primer sequence	Amplicon length (bp)	PCR cycle	Primer source
12S rRNA	12Sa	AAACTGGGATTAGATACCCCACTAT	370–381	94 °C (30sec),48 °C (45sec),72 °C (1min),35 cycles	Kocher et al. (1989)
	12Sb	GAGGGTGACGGGCGGTGTGT			
16S rRNA	16SL1	CGCCTGTTTAACAAAAACAT	449–455	94 °C (1min),47 °C (45sec),72 °C (1min),40 cycles	Palumbi et al. (1991)
	16SH1	CCGGTCTGAACTCAGATCACGT			
cytb	Cytb1	CCATCCAACATCTCAGCATGATGAAA	307	94 °C (35sec),45–46 °C (35sec),72 °C (1min 30sec),30 cycles	Kocher et al. (1989)
	Cytb2	CCCTCAGAATGATATTTGTCCTCA			
cmos	FUF	TTTGGTTCKGTCTACAAGGCTAC	415	94 °C (30sec),53 °C (45sec),72 °C (1min 30sec),35 cycles	Gamble et al. (2008)
	FUR	AGGGAACATCCAAAGTCTCCAAT			

### Phylogenetic analyses

The dataset was partitioned by gene. Models of sequence evolution were assessed for each partition by Partition Finder v.1.1 (Lanfear et al. 2012) with the following settings: branch lengths linked, models available in BEAST evaluated, model selection based on BIC. The models identified as most suitable were as follows: GTR+I+Γ for the 12S, 16S, and ND4, SYM+I+Γ for cytb and HKY+I+Γ for cmos. As outgroups, we used 21 species representing the genera *Rhachisaurus*, *Gymnophthalmus*, *Alopoglossus*, *Riolama*, *Ecpleopus*, and *Bachia* that are known to be closely related to the cercosaurine genera but not being part of the subfamily (Pyron et al. 2013; Kok 2015). The outgroup species are also listed in Suppl. material 1: Table S1. In total the dataset consisted of 357 samples, of which 26 represented the outgroup taxa.

Phylogenetic analyses were conducted by means of maximum likelihood (ML) and Bayesian inference (BI). The ML analysis was conducted using RAxML-HPC2 v.8.2.9 (Stamatakis 2014) with a heuristic search that included 100 random addition replicates and 1000 thorough bootstrap pseudoreplications. We applied the GTR+CAT model to all partitions as the CAT model has been shown to be a faster and computationally less demanding alternative to the Γ model (Stamatakis 2006; Stamatakis et al. 2008). We skipped the +I parameter because the 25 default rate categories of the CAT model account for potentially invariant sites (Stamatakis 2006).

The Bayesian analyses were conducted using MrBayes v.3.2 (Ronquist et al. 2012) and BEAST 2.4.5 (Bouckaert et al. 2014). MrBayes settings were the following: GTR as the preferred substitution model for the mtDNA genes and HKY for cmos, invariable proportions of among-site rate variation and a gamma-distributed rate parameter applied, ploidy set to haploid for the mtDNA genes, four parallel runs ran each with four chains, number of generations set to 10^8^ with a 10^5^ sampling frequency, 10% of trees discarded as burnin. Stationarity was confirmed by the value of average standard deviations of the split frequencies being lower than 0.01. Convergence of the four runs was confirmed by the values of PSRF (potential scale reduction factor) reaching 1.00. Estimated parameter values were inspected and a 50% majority-rule consensus tree was generated in MrBayes.

The second Bayesian analysis was run in BEAST. In order to avoid over-parameterisation caused by the large size of the dataset, we applied the HKY model for all partitions instead of the GTR as preferred by PartitionFinder. The Γ parameter was selected to have four categories and shape estimated. We applied the Yule process tree prior with uniformly distributed birth rate (lower: 0, upper: 1000) and an independent relaxed uncorrelated lognormal clock prior for each partition. Ambiguities in the cmos alignment coded by the IUPAC ambiguity codes were accounted for. Clock rates were set to have lognormal distributions with the mean = 1 and st. dev. = 1.25 for the mtDNA genes and mean = 0 and st. dev. = 1.0 for the cmos relative to the first partition of the concatenated alignment, which was the 12S. Standard deviation of the clock parameter (among-lineage rate heterogeneity) was for all partitions estimated with an exponential distribution with the mean = 1. Four independent runs were made, each for 2.5×10^8^ MCMC generations and parameters logged every 10^5^ generation. 10% of sampled trees were discarded from each analysis as burnin. Stationarity, convergence of the runs, and effective sample sizes (ESS) of all parameters were inspected in Tracer v.1.5 (Rambaut and Drummond 2007). Post-burnin posterior trees were combined using LogCombiner v.2.4.5 and the maximum clade credibility (MCC) tree with mean node heights was identified and posterior probabilities calculated using TreeAnnotator v.1.7.5.

All phylogenetic analyses were run through the CIPRES Science Gateway (Miller et al. 2010). Tree nodes were considered strongly supported when they received ML bootstrap support ≥ 70% and posterior probability (pp) values inferred by the two BI analyses ≥ 0.95.

Genetic distances between the clades of cercosaurines were calculated for all genetic markers analysed except cytb using MEGA 6 (Tamura et al. 2013) with the pairwise deletion option selected.

### Morphological characters

The format of the descriptions and terminology of the morphological characters follow mostly Oftedal (1974), Chávez et al. (2017), and Sánchez-Pacheco et al. (2017b). Specimens were fixed in 96% and stored in 70% ethanol. Sex and maturity of specimens were identified through dissection of gonads. Specimens with SVL ≤ 30.2 mm were considered juveniles. The following metric characters were taken using a digital caliper and dissecting microscope (to the nearest 0.1 mm):

**SVL** snout-vent length – distance from the snout tip to cloaca;

**HL** head length – distance from the snout tip to the angle of jaw;

**HW** head width – greatest width of the head;

**HD** head depth – greatest depth of the head;

**TL** tail length – distance from cloaca to the tail tip, if original;

**E–N** eye-snout distance – straight distance from the snout tip to anterior corner of eye;

**FLL** forelimb length – from axilla to tip of distal claw;

**HLL** hindlimb length – from groin to tip of distal claw;

**AGD** axilla-groin distance – distance between limbs;

**hemipenis length** distance from hemipenial base to distal margin of hemipenial lobes.

Meristic and qualitative pholidotic characters were counted and evaluated as follows: number of supralabials from the rostral to the mouth corner, last labial defined by its considerably larger size compared with the posteriorly adjacent shields; dorsal scales by the number of transverse rows of dorsal scales from the third row behind the interparietal to the level of the rear edge of the hindlimb; ventral scales, the number of transverse rows of ventral scales (from collar to the anterior row of anal scales); lateral scales, the number of longitudinal rows of considerably smaller lateral scales lying between larger dorsal and ventral scales at midbody; scales around midbody; preanal plates are the number of large plates in the posterior row of anal scales; number of lamellae under Finger IV including the number of single and divided lamellae (left/right, lamellae divided into segments counted as one individual lamella); number of lamellae under Toe IV refers to the number of single and divided lamellae (left/right, lamellae divided into segments counted as one individual lamella); number of preanal pores (left/right).

Description of colouration in life was based on field notes and photographs. Collection acronyms are: **MUSM** Museo de Historia Natural Universidad Nacional Mayor de San Marcos, Lima, Peru; **NMP6V** National Museum Prague, Prague, Czech Republic; **SMF** Senckenberg Forschungsinstitut und Naturmuseum, Frankfurt, Germany. Field number codes are: **IWU** Illinois Wesleyan University; **JCM** Juan Carlos Cusi collection. Threat status was evaluated using the IUCN criteria (2016). High-resolution versions of photographs presented in this article and additional pictures of the type specimens have been uploaded to MorphoBank (project number: 3136; http://www.morphobank.org) where they are available for download.

### Drawings and maps

All drawings were made by the senior author using a stereomicroscope and a camera lucida. Maps were made with QGIS (Quantum GIS Development Team 2014).

**Figure 1.**
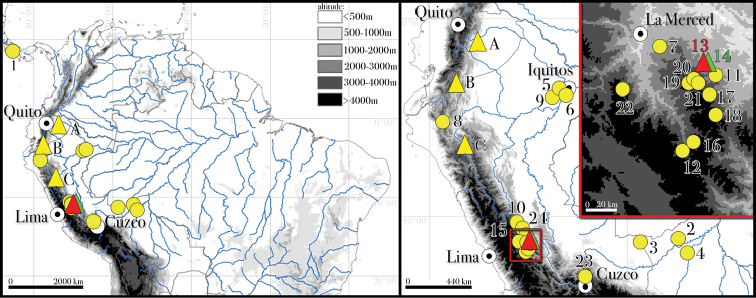
Map showing localities of samples newly sequenced for this study. Locality numbers correspond to those in Table 1. Localities of the new genus described here, *Selvasaura* gen. n., are marked with triangles; red triangle indicates the type locality of its type species, *S. brava* sp. n.; green triangle locality of paratypes MUSM 32718 and NMP6V 75655; yellow triangles localities published by Torres-Carvajal et al. (2016): **A** Provincia de Napo, Wildsumaco Wildlife Sanctuary, Ecuador **B** Provincia de Zamora Chinchipe, El Pangui, Ecuador **C** region San Martin, Provincia Mariscal Cáceres, Laurel, Peru. White circles denote major cities.

## Results

### Phylogenetic analyses

**Figure 2.**
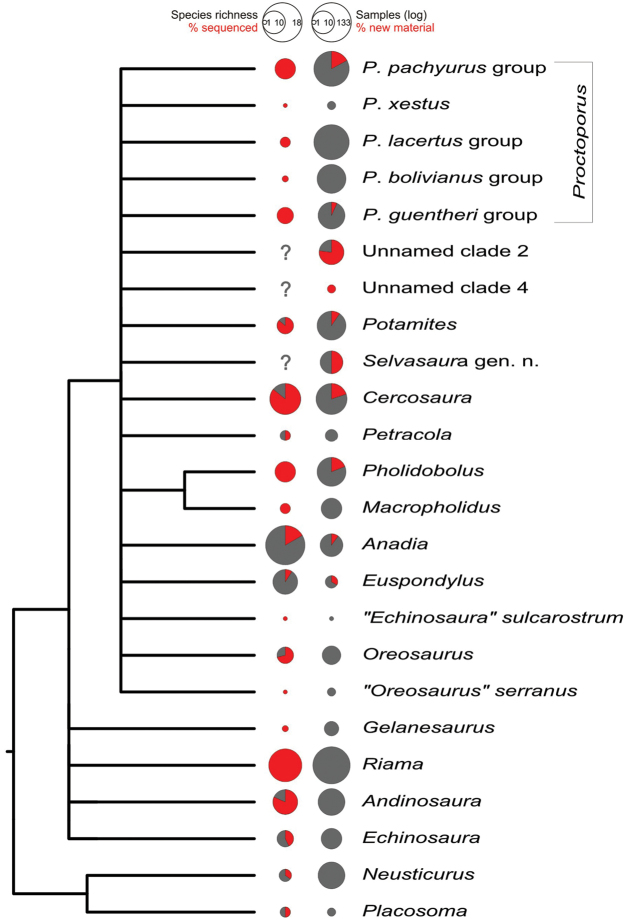
Phylogenetic tree showing relationships between cercosaurine genera or, in cases when genera were not recovered as monophyletic, their major lineages. The tree is a strict consensus tree based on the results of three analytical approaches undertaken: ML, MrBayes, BEAST. The 24 lineages shown were supported in all three phylogenetic analyses. Relationships between genera are shown as dichotomies only for nodes that were strongly supported in all three analyses; otherwise, nodes were collapsed into polytomies to emphasise how little we can tell about the phylogeny of the subfamily Cercosaurinae with the data currently available. Outgroups are not depicted. For a tree that shows variability within genera see Fig. 3, for full trees see Fig. S1–S3. The pie charts on the right show i) species richness of the genera indicated by circle size with the proportion of species included in the analyses highlighted in red (left column), and ii) number of samples (log scale) available for each genus indicated by circle size with the proportion of material newly sequenced in this study in red (right column).

**Figure 3:**
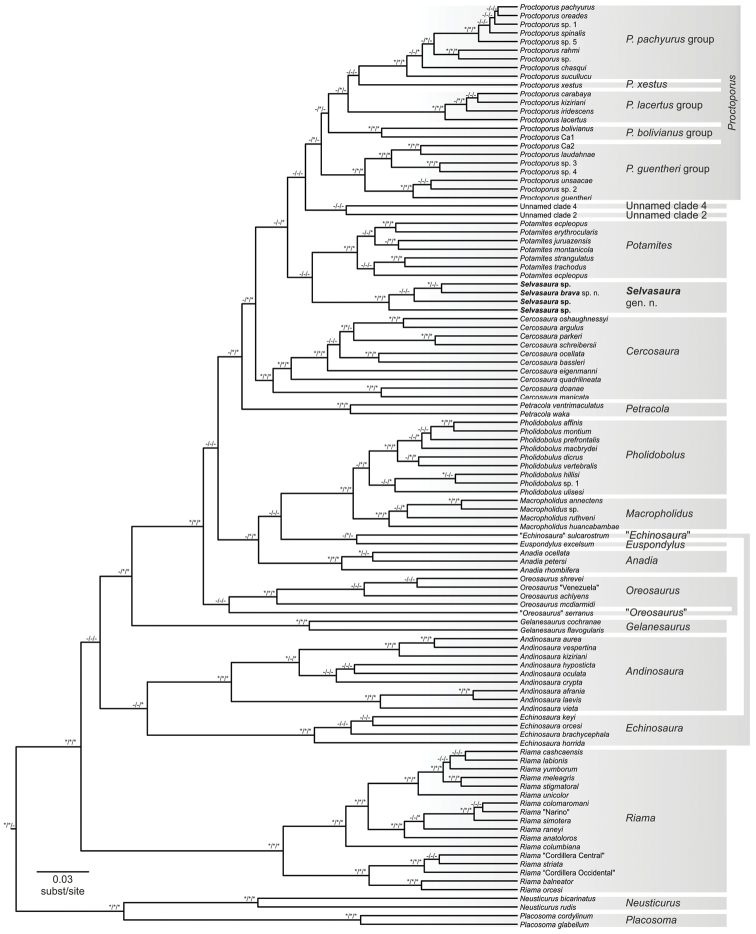
. Maximum clade credibility tree for 107 species (both described and candidate) of the subfamily Cercosaurinae from the BEAST analysis. The dataset for the analysis contained 357 samples with most species being represented by multiple samples, but for visual purposes only one sample was retained for each species in this tree. Nodal support is shown in the ML/MrBayes/BEAST order; supported nodes are marked with asterisks, unsupported with dashes. Monophyletic groups at the genus level are highlighted by grey rectangles. Vertical grey bars connect species that supposedly belong to one genus, but whose monophyly was not supported in any of the phylogenetic analyses: the genera *Proctoporus*, *Echinosaura*, and *Oreosaurus*. Outgroups are not depicted. For a full BEAST tree see Fig. S3.

All three analyses performed here resulted in topologies concordant with previous studies (Torres-Carvajal et al. 2016, Sánchez-Pacheco et al. 2017b). The subfamily Cercosaurinae was monophyletic, although the pp support from the BEAST analysis did not exceed the 0.95 threshold (ML bootstrap: 100; MrBayes pp: 1; BEAST pp: 0.92; nodal support values in the same order hereafter; Suppl. material 1: Figs S1–S3). Most of the cercosaurine genera were strongly supported in all the analyses: *Anadia* (100, 1, 1); *Andinosaura* (86, 1, 1); *Cercosaura* (78, 1, 1); *Euspondylus* (98, 1, 1); *Gelanesaurus* (100, 1, 1); *Macropholidus* (92, 1, 1); *Neusticurus* (98, 1, 1); *Petracola* (93, 1, 1); *Pholidobolus* (63, 0.97, 0.96); *Placosoma* (100, 1, 1); *Potamites* (93, 1, 1); *Riama* (100, 1, 1). Two undescribed clades recovered by Torres-Carvajal et al. (2016) and referred to as Unnamed clade 2 and 3 were also strongly supported here: Unnamed clade 2 (100, 1, 1), Unnamed clade 3 (96, 1, 1), the latter being part of the ramification of the new genus described herein. Additionally, we identified yet another clade that may deserve recognition as an independent genus and that we term tentatively Unnamed clade 4 (support 91, 1, 1) and whose phylogenetic affinities to the other genera remained unclear due to low support (Figs 2, 3). Unnamed clade 4 consisted of two samples collected by EL and collaborators in cloud forests in the surroundings of Alfamayo (Region of Cuzco, Peru) and in a montane forest close to the National Park Yanachaga-Chemillén (Region Pasco, Peru).

Monophyly was not supported for three of the described genera (Fig. 3). First, one species of the genus *Echinosaura*, *E. sulcarostrum*, did not cluster with the remaining four species of the genus included in the dataset and which formed a clade (100, 1, 1; as previously found by Torres-Carvajal et al. 2016). It was instead topologically closest to *Euspondylus*, although support of this sister relationship was low in two of the analyses (35, 0.96, 0.92). Second, monophyly of the recently resurrected genus *Oreosaurus* (Sánchez-Pacheco et al. 2017b) was also questionable. Although the topology of the BEAST tree shows all *Oreosaurus* species to form one group, monophyly of this group was not supported in any of the analyses and the phylogenetic position of *O. serranus* was unstable across the analyses. The other four *Oreosaurus* species that were included in the dataset formed a clade (80, 1, 1). Third, in concordance with Torres-Carvajal et al. (2016), but contrary to Goicoechea et al. (2012) and Sánchez-Pacheco et.al. (2017b), monophyly of *Proctoporus* was found to be questionable as the genus was supported only in the MrBayes analysis while it received no support in the ML and BEAST analyses (13, 0.95, 0.51). The *Proctoporus* species formed five well supported groups: i) a clade of *P. pachyurus*, *P. oreades*, *P. spinalis*, *P. rahmi*, *P. chasqui*, *P. sucullucu*, and three yet undescribed species (support 88, 1, 1; termed *Proctoporus pachyurus* group); ii) a clade comprising a single species, *P. xestus* (98, 1, 1); iii) a clade of *P. carabaya*, *P. kiziriani*, *P. iridescens*, *P. lacertus* (98, 1, 1; termed *Proctoporus lacertus* group); iv) a clade of *P. bolivianus* and one undescribed species (93, 1, 1; termed *Proctoporus bolivianus* group); v) a clade of *P. unsaacae*, *P. guentheri*, *P. laudahnae*, and four undescribed species (96, 1, 1; termed *Proctoporus guentheri* group). Mutual relationships between these five *Proctoporus* groups as well as their relationships to the other cercosaurine genera remained unresolved.

Higher-level relationships between the cercosaurine genera were difficult to infer for the generally low node support at this phylogenetic depth. Only a few clades could be identified that were common to the three different phylogenetic analyses undertaken (Fig. 2). In all analyses, *Neusticurus* was sister to *Placosoma* (100, 1, 1) and the clade of these two was sister to all the remaining genera of Cercosaurinae (76, 0.99, 1). Of the remaining genera, only the sister pair *Pholidobolus*/*Macropholidus* was recovered in all analyses with strong support (100, 1, 1) and a large clade comprising the five *Proctoporus* groups, *Potamites*, *Cercosaura*, *Petracola*, *Pholidobolus*, *Macropholidus*, *Anadia*, *Euspondylus*, “*Echinosaura*” *sulcarostrum*, both *Oreosaurus* lineages, Unnamed clades 2 and 4, and the new genus described herein (100, 1, 1). Otherwise, no other genera clustered into clades that would be supported by all three phylogenetic approaches.

**Figure 4.**
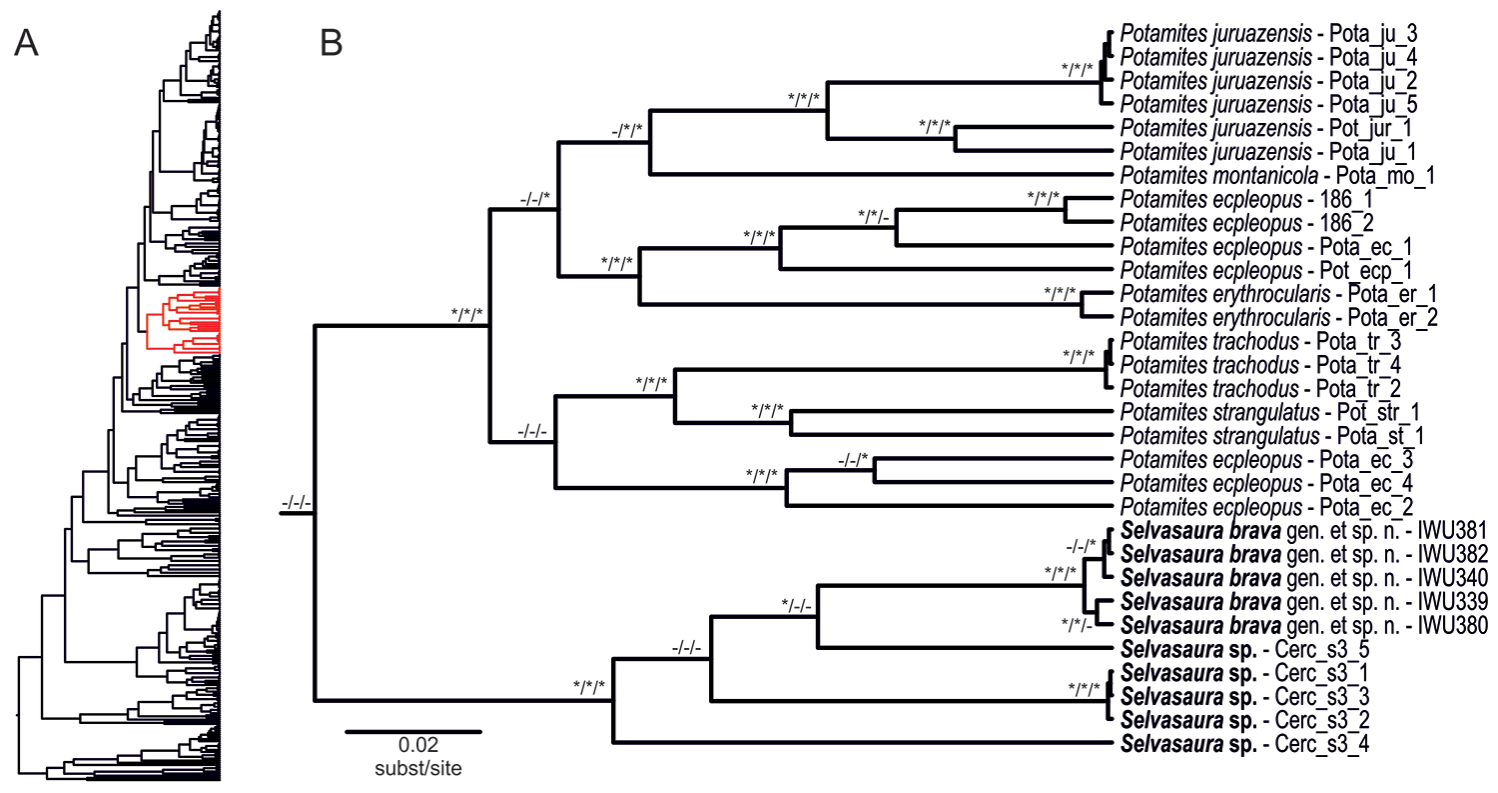
**A** Maximum clade credibility tree of Cercosaurinae based on the BEAST analysis with the position of *Selvasaura* gen. n. and *Potamites* highlighted in red **B** A close-up of the red part of the tree in the left showing the phylogenetic relationships between and within *Selvasaura* gen. n. and *Potamites*. Nodal support is shown in the ML/MrBayes/BEAST order; supported nodes are marked with asterisks, unsupported with dashes. Note that the basal node in the inset is not supported and that the sister relationship of the two genera may not be real.

Phylogenetic affinities of the new genus described herein to the other cercosaurine genera remained unresolved (Fig. 3). Although it was reconstructed as a sister lineage to the genus *Potamites* in all analyses, the topology was not supported in any of them (47, 0.55, 0.89). Within the genus, all analyses unambiguously identified four well genetically differentiated lineages (Fig. 4), albeit their mutual relationships remained unresolved due to the lack of nodal support. The first was a cluster of five samples from the PPPF, Peru that represent the new species described below, the second of one sample from El Pangui, Zamora-Chinchipe Province, Ecuador (voucher QCAZ 12891; sample code Cerc_s3_5), the third of three samples from Laurel, Mariscal Cáceres Province, Peru (vouchers CORBIDI 15117–19; sample codes Cerc_s3_1–3), and the fourth of one sample from Wildsumaco Wildlife Sanctuary, Napo Province, Ecuador (voucher QCAZ 12798; sample code Cerc_s3_4) (Fig. 1). The three latter lineages were published by Torres-Carvajal et al. (2016), who also found the weekly supported sister relationship between *Potamites* and the new genus.

### Morphological characters

The examined morphological characters were used for comparisons with other genera and for the formal descriptions of the new genus and species provided below.

## Taxonomy

### Family Gymnophthalmidae Fitzinger, 1826

#### Subfamily Cercosaurinae Gray, 1838

##### Genus Selvasaura gen. n.

Taxon classificationAnimaliaSquamataGymnophthalmidae

http://zoobank.org/71A0F024-36F5-4420-BEEF-7222AE7B9534

Unnamed clade 3 (in Torres-Carvajal et al. 2016)

###### Type species.

*Selvasaura brava* sp. n.

###### Diagnosis.

Phenotypic synapomorphies are not known for this genus. Morphologically, *Selvasaura* gen. n. can be distinguished from all other genera of Cercosaurinae by the combination of the following characters: lower palpebral disc transparent, not divided (divided in *Andinosaura*, *Euspondylus*, *Gelanesaurus*, *Oreosaurus*, *Petracola*, *Riama*, and most *Anadia* and *Placosoma* species; opaque in *Pholidobolus*); dorsal scales slightly rugose (smooth in *Anadia*; keeled in *Cercosaura*; strongly keeled and tuberculate in *Echinosaura*, *Gelanesaurus*, *Neusticurus*, *Potamites*; minute tubercles on posterior dorsal scales in *Placosoma*); lateral scales distinctly smaller than dorsal scales (lateral scales not distinctly reduced in size in *Macropholidus*); lateral scales adjacent to ventrals non-granular (granular in *Proctoporus*) (see e.g., Oftedal 1974; Cadle and Chuna 1995; Altamirano-Benavides et al. 2013; Kok et al. 2013; Torres-Carvajal and Mafla-Endara 2013; Echevarría et al. 2015; Borges-Nojosa et al. 2016; Chávez et al. 2017; Sánchez-Pacheco et al. 2017b). Genetically, the genus is differentiated from the other cercosaurines by distances given in Table 3 and 4.

**Table 3.** Mean uncorrected genetic distances (*p*-distances, in %) between cercosaurine genera or their monophyletic clades if the genus’ monophyly was not supported. Below diagonal are values based on the 12S alignment, above on the 16S. Values for *Selvasaura* gen. n. are in bold.

	*Anadia*	*Andinosaura*	*Cercosaura*	*Echinosaura*	“*Echinosaura*” *sulcarostrum*	*Euspondylus*	*Gelanesaurus*	*Macropholidus*	*Neusticurus*	*Oreosaurus*	“*Oreosaurus*” *serranus*	*Petracola*	*Pholidobolus*	*Placosoma*	*Potamites*	*Proctoporus bolivianus* group	*Proctoporus guentheri* group	*Proctoporus lacertus* group	*Proctoporus pachyurus* group	*Proctoporus xestus*	*Riama*	*Selvasaura* gen. n.	Unnamedclade 2	Unnamedclade 4
*Anadia*		9.02	7.82	8.8	7.37	5.6	7.22	5.95	9.31	7.33	7.17	8.32	6.31	11.76	6.86	6.79	6.86	6.21	6.33	6.73	9.13	**6.19**	6.46	5.66
*Andinosaura*	10.44		9.15	8.42	8.97	8.41	8.32	8.91	10.82	8.6	9.28	9.16	8.64	12.16	9.68	7.67	8.57	7.59	7.89	8.18	8.45	**8.61**	9.16	9.19
*Cercosaura*	9.88	11.88		10.2	8.06	6.67	8.72	7.89	11.16	7.05	6.93	6.71	6.9	11.77	7.16	5.33	5.96	5.06	5.03	5.81	9.08	**5.66**	6.1	6.02
*Echinosaura*	13.77	13.79	12.84		9.65	9.38	7.43	8.92	10.91	9.97	9.52	9.77	9.61	11.2	9.3	9.54	9.53	9.21	9.31	8.91	8.06	**9.29**	9.61	9.19
“*Echinosaura*” *sulcarostrum*	9.21	12.82	10.74	12.65		5.97	8.39	7.57	10.25	7.42	7.22	6.44	7.43	11.84	7.9	6.68	6.94	5.92	6.21	6.12	8.84	**6.84**	6.43	7.11
*Euspondylus*	7.42	9.59	8.73	11.61	6.06		7.82	4.74	9.2	5.75	5.54	6.58	4.38	10.8	5.55	4.59	4.69	3.86	4.64	4.83	7.35	**5.12**	3.72	4.56
*Gelanesaurus*	11.74	12.76	12.99	14.71	13.13	11.18		7.64	10.07	7.65	7.18	8.32	8.23	11.34	7.62	7.56	7.75	6.97	7.04	6.85	6.85	**6.82**	7.87	6.85
*Macropholidus*	8.97	8.71	8.54	13.1	7.53	7.09	13.03		9.85	6.41	6.07	8.42	3.05	12.93	6.84	6.47	6.62	5.64	6.39	6.61	8.22	**6.27**	5.86	5.49
*Neusticurus*	11.04	14.67	13.66	14.33	14.24	11.41	13.6	11.89		9.9	10.72	10.63	9.45	10.43	10.4	9.41	9.71	9.11	9.26	9.75	10.06	**9.95**	9.78	10.19
*Oreosaurus*	8.48	11.33	8.74	12.48	9.57	6.65	11.4	7.64	13.04		6.42	6.56	6.2	12.04	7.09	5.39	5.55	5.08	5.27	5.8	8.75	**5.58**	5.51	5.9
“*Oreosaurus*” *serranus*	10.06	11.62	10.57	14.1	12	9.03	12.54	8.2	14.86	8.13		6.87	6.4	11.48	6.93	5.97	5.66	4.68	5.02	5.44	8.97	**5.79**	5.51	5.42
*Petracola*	8.27	11.82	9.11	13.42	10.27	7.19	11.94	8.44	13.95	8.06	8.52		7.6	11.84	7.16	5.74	5.36	4.85	5.06	4.95	8.58	**6.27**	6.2	5.93
*Pholidobolus*	8.59	9.37	8.93	12.61	7.86	6.16	11.7	4.31	11.85	6.99	8.62	8.14		11.86	6.6	5.86	5.92	5.11	5.8	6.21	7.85	**5.81**	5.4	5.91
*Placosoma*	16.73	18.61	17.04	17.98	18.92	16.9	17.68	16.65	15.87	16.36	18	16.84	17.47		11.27	11.12	11.54	10.99	11.27	10.91	11.06	**11.81**	11.37	11.89
*Potamites*	8.44	10.17	7.87	12.39	7.08	6.86	12	7.77	12.81	6.91	8.86	6.69	7.55	15.61		6.1	5.55	4.61	4.86	5.8	8.29	**4.82**	5.51	4.96
*Proctoporus bolivianus* group	9.39	12.04	7.68	12.08	9.37	7.63	12.5	8.39	14.53	7.62	10.5	6.28	8.1	17.38	5.71		4.2	3.2	3.15	4.25	8.32	**4.33**	4.2	4.36
*P. guentheri* group	8.86	11.14	8.4	12.55	8.02	7.67	12.41	8.09	12.98	7.24	8.92	5.66	8.17	16.17	6.26	5.26		2.92	3.6	4.97	8.27	**4.75**	4.34	4.09
*P. lacertus* group	7.83	10.72	7.67	12.01	7.95	5.83	11.25	7.3	13.51	6.3	8.98	4.85	6.92	17.47	5.03	3.96	3.99		2.13	3.3	7.42	**3.75**	3.61	2.73
*P. pachyurus* group	7.91	10.48	7.4	11.66	7.94	7.15	11.44	6.73	12.95	6.52	8.46	5.28	6.79	16.29	5.17	4.74	4.32	3.4		3.79	8.09	**3.7**	3.97	3.19
*P. xestus*	9.16	12.03	8.48	11.63	8.78	7.21	12.39	7.89	13.8	8.4	10.48	7.1	7.78	18.72	6.57	5.61	5.78	4.41	4.45		7.76	**4.34**	4.53	3.6
*Riama*	10.36	11.13	10.59	11.91	11.51	9.31	13.05	9.13	13.67	9.37	11.46	10.18	9.1	18.55	9.08	9.13	9.11	8.29	7.93	9.48		**8.55**	8.01	8.59
***Selvasaura* gen. n.**	**7.88**	**9.86**	**7.37**	**11.86**	**8.46**	**7.04**	**11.92**	**7.01**	**12.42**	**7.13**	**8.83**	**5.75**	**7.58**	**15.78**	**5.37**	**5.23**	**4.75**	**4.21**	**3.8**	**5.35**	**8.22**		**4.35**	**3.89**
Unnamed clade 2	8.57	11.55	8.05	11.45	9.16	7.44	11.78	7.03	13.43	7.24	8.82	5.76	7.92	16.52	5.95	5.39	4.88	4.17	3.79	5.75	8.56	**4.81**		3.65
Unnamed clade 4	9.47	11.69	8.15	13.04	9.54	8.05	12.37	8.7	14.94	7.82	8.15	6.52	8.76	15.31	5.47	6.57	6.08	5.52	5.08	7.47	10.31	**5.82**	6.23	

**Table 4.** Mean uncorrected genetic distances (*p*-distances, in %) between cercosaurine genera or their monophyletic clades if the genus’ monophyly was not supported. Below diagonal are values based on the ND4 alignment, above on the cmos. Values for *Selvasaura* gen. n. are in bold. Note that compared to Table 3 some clades are missing here because they did not have the ND4 and cmos sequences available (“*Echinosaura*” *sulcarostrum*, Unnamed clade 4).

	***Anadia***	***Andinosaura***	***Cercosaura***	***Echinosaura***	***Euspondylus***	***Gelanesaurus***	***Macropholidus***	***Neusticurus***	***Oreosaurus***	“***Oreosaurus*” *serranus***	***Petracola***	***Pholidobolus***	***Placosoma***	***Potamites***	***Proctoporus bolivianus* group**	***Proctoporus guentheri* group**	***Proctoporus lacertus* group**	***Proctoporus pachyurus* group**	***Proctoporus xestus***	***Riama***	***Selvasaura* gen. n.**	**Unnamedclade 2**
*Anadia*		3.73	5.61	3.57	2.26	4.23	1.26	4.31	3.2	3.03	3.21	1.49	6.85	2.91	3.96	4.37	4.02	3.65	3.64	4.59	**3.15**	4.21
*Andinosaura*	23.1		7.33	2.73	4.49	4.68	3.65	3.63	4.95	5.04	5.08	3.66	6.69	4.68	5.66	5.85	5.73	5.49	5.35	3.33	**4.91**	5.75
*Cercosaura*	20.8	22.3		6.82	4.86	7	4.98	6.76	5.59	5.63	5.5	5.09	9.52	3.28	3.6	4.49	3.7	3.39	3.3	6.57	**3.43**	4.7
*Echinosaura*	25.3	23.5	24.6		4.02	4.15	3.09	2.74	4.45	4.5	4.32	3.17	6.21	4.13	5.19	5.35	5.31	4.92	4.92	3.05	**4.34**	5.08
*Euspondylus*	17.5	21.2	19.7	25		4.18	1.22	4.22	2.85	2.68	2.83	1.33	7.46	2.73	3.79	4.2	3.85	3.11	3.48	4.41	**2.99**	4.06
*Gelanesaurus*	23.8	23.3	23.1	26.2	24.4		3.76	4.41	4.61	4.41	4.73	3.85	7.19	4.28	5.38	5.12	5.41	5.07	5.07	4.37	**4.54**	5.45
*Macropholidus*	19.8	22.5	20.6	26.3	17.7	24.6		3.79	2.67	2.67	2.86	0.44	6.85	2.39	3.42	3.86	3.48	3.16	3.11	4.06	**2.62**	3.7
*Neusticurus*	25	23.3	23.1	24.9	24	24.2	26		4.51	4.81	4.9	3.83	5.6	4.24	5.38	5.52	5.46	4.72	5.07	3.08	**4.04**	5.45
*Oreosaurus*	20.8	22.9	21.3	25.6	20.2	23.3	21.3	24.1		3.61	3.67	2.8	7.74	3.14	4.18	4.57	4.24	3.76	3.87	4.83	**3.5**	4.62
“*Oreosaurus*” *serranus*	22.2	24.6	21.4	26.3	22.9	23.8	23.5	25.8	21.3		2.84	2.86	7.49	3.36	4.32	4.8	4.36	4.09	4.01	4.45	**3.58**	4.41
*Petracola*	20.4	21.3	17.7	24.1	20.9	23.2	19.8	22.4	20.6	22		2.89	8.04	2.84	3.7	4.23	3.85	3.54	3.47	4.16	**3.01**	3.65
*Pholidobolus*	19.4	22.4	20	26.2	17.5	24.1	17.7	25.1	20.5	22.7	20.6		7.19	2.49	3.43	3.94	3.5	3.24	3.12	4.08	**2.68**	3.74
*Placosoma*	25.7	25.2	24.3	25.6	23.4	25.2	24.8	22.3	24.4	25.8	23.7	25.1		7.42	8.71	8.32	8.75	7.96	8.4	5.84	**7.33**	8.56
*Potamites*	21	23.2	19.7	25.6	20.7	24.1	21.7	23.1	21.3	21.4	19.9	20	25.2		1.49	1.99	1.6	1.19	1.19	3.89	**0.77**	2.33
*Proctoporus bolivianus* group	20.7	22.2	19.1	24.7	19.6	23	20.4	22.9	20.2	19.2	18.2	19.7	25.1	19.2		2.69	1.23	1.8	0.84	4.93	**1.54**	3.06
*Proctoporus guentheri* group	19.7	21.9	18.7	24.2	19.6	22.9	19.9	23.4	20.5	19.4	18.6	19.4	24.8	18.1	16.5		2.77	2.43	2.38	5.13	**1.99**	2.93
*Proctoporus lacertus* group	20.6	23.1	19.5	25	19.6	23.2	20.8	24.5	20.1	20.8	19.4	19.5	25.2	18.3	16.8	15.3		1.82	0.41	5.03	**1.63**	3.14
*Proctoporus pachyurus* group	19.6	22.2	18.4	24.7	19.3	21.9	20.5	22.3	19.1	20.1	17.9	19.1	24.7	17.8	15.9	15.7	15.5		1.42	4.35	**0.89**	2.78
*Proctoporus xestus*	22.7	23.5	21.1	25.7	20.9	24.5	23.8	25.5	21.2	21	20.6	21.8	27.3	20.9	20	18.6	19	18.2		4.64	**1.23**	2.77
*Riama*	24.1	23.6	24.4	25.8	23.3	22.7	24.3	24.6	23.2	24.9	23.9	24.1	25	24.2	24.1	23.3	23.9	23	25.4		**3.69**	4.74
***Selvasaura* gen. n.**	**20.7**	**22.4**	**19.8**	**24.4**	**19.6**	**23**	**21.3**	**23.6**	**21.4**	**19.7**	**20.1**	**19.8**	**24.7**	**19.5**	**18.7**	**17.5**	**18.4**	**17.3**	**19.5**	**23.7**		**2.27**
Unnamed clade 2	21.3	20.8	19.9	24.4	17.8	23.2	19.6	23.3	20.3	19.4	19	20.2	23.7	20.3	17.4	18	18.6	18.6	19.6	23.7	**19**	

###### Definition.

(1) head shields smooth; (2) frontoparietal and parietal shields paired; (3) frontonasal, frontal and interparietal shields single; (4) prefrontal shields present; (5) lower palpebral disc transparent, not divided; (6) loreal shield present; (7) scale organs on labials present; (8) anteriormost supraocular and anteriormost superciliary shields fused; (9) dorsal surface of the tongue covered by scale-like papillae; (10) nuchal scales smooth; (11) dorsal scales rectangular, slightly rugose; (12) ventral scales squared to rectangular, smooth; (13) limbs pentadactyl, digits clawed; (14) femoral pores present in males, absent in females; (15) hemipenial lobes large, distinct from the hemipenial body.

###### Content.

*Selvasaura brava* sp. n. and undescribed species of Unnamed clade 3 (sensu Torres-Carvajal et al. 2016) whose formal descriptions are underway (see Torres-Carvajal et al. 2016).

###### Distribution.

Peru: Región Junín, Provincia de Chanchamayo, Pui Pui Protected Forest (*Selvasaura brava* sp. n.); Región San Martin, Provincia Mariscal Cáceres, Laurel (Cercosaurinae sp. 3; Torres-Carvajal et al. 2016). Ecuador: Provincia de Zamora Chinchipe, El Pangui (Cercosaurinae sp. 3; Torres-Carvajal et al. 2016); Provincia de Napo, Wildsumaco Wildlife Sanctuary (Cercosaurinae sp. 3; Torres-Carvajal et al. 2016).

###### Etymology.

The generic name *Selvasaura* is derived from the Spanish noun ‘*selva*’ (forest) and the Greek noun *σαύρα* (lizard; *saura* is the feminine form) and refers to the habitat (montane rainforest) of the type species.

##### Selvasaura brava sp. n.

Taxon classificationAnimaliaSquamataGymnophthalmidae

http://zoobank.org/88FAD0FE-8FBC-41BD-BCD2-334715157340

###### Holotype.

**Figure 5.**
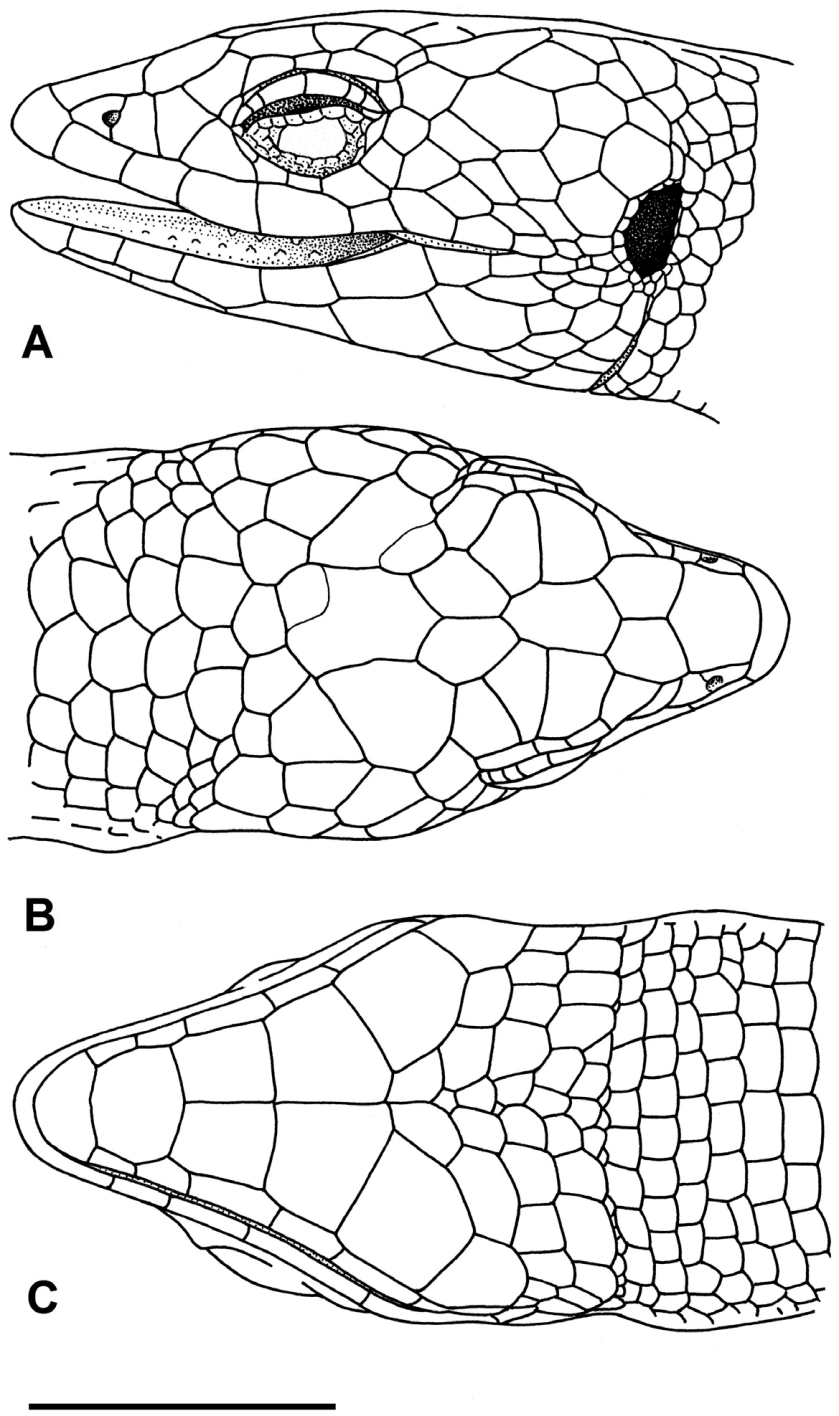
Drawing of the head of the holotype of *Selvasaura brava* sp. n. (MUSM 32738). **A** lateral, **B** dorsal **C** ventral view. Scale bar: 5 mm. Drawing by J. Moravec.

**Figure 6.**
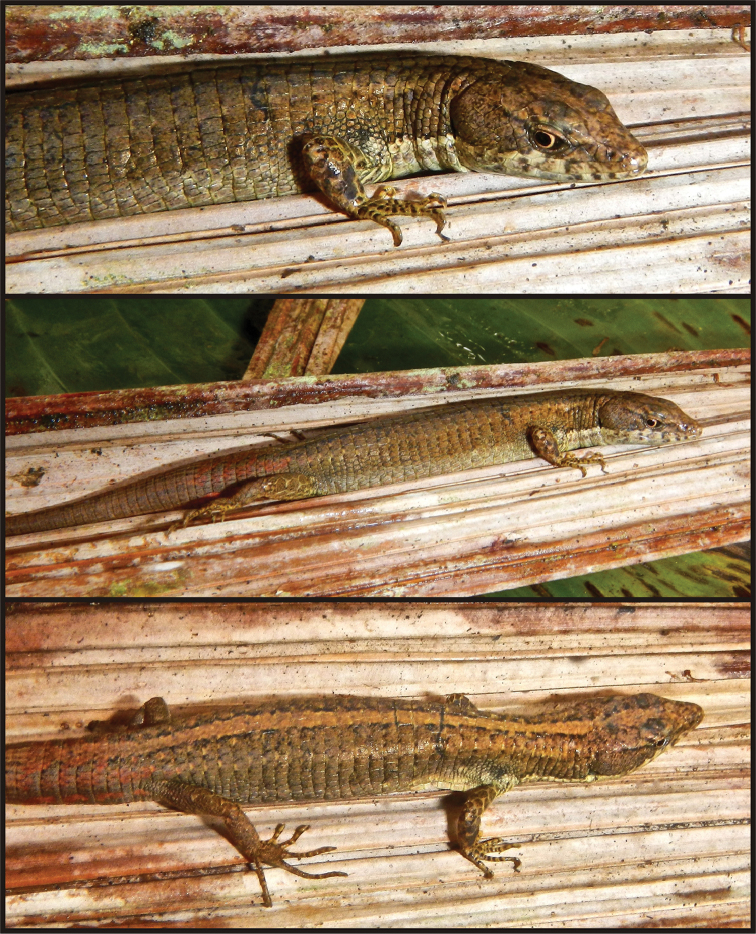
Holotype of *Selvasaura brava* sp. n. (MUSM 32738) in life. Photographs by E. Lehr.

(Figs 5, 6). MUSM 32738 (sample code IWU 381; MorphoBank pictures: M485668–M485671), an adult male from the border of the Pui Pui Protected Forest (11.211S, 74.958W; WGS84), 1700 m elevation, Distrito Pichanaqui, Provincia Chanchamayo, Región Junín, Peru, collected on 19 May 2014 by Edgar Lehr and Jiří Moravec.

###### Paratypes.

**Figure 7.**
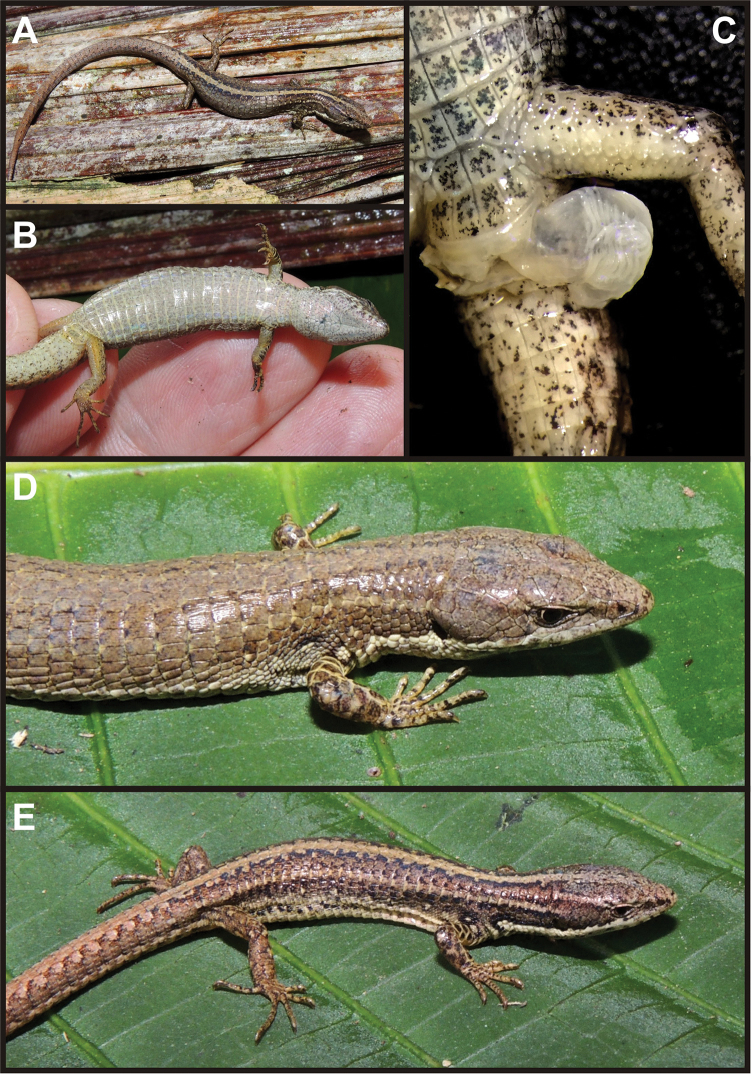
Paratypes of *Selvasaura brava* sp. n. Dorsal (**A**) and ventral (**B**) view of adult male (NMP6V 75653) with a detail of an everted hemipenis (**C**) **D** adult female (MUSM 32718) **E** – juvenile (NMP6V 75655). Note the generally uniform colouration of the female compared to the male and juvenile specimens. Photographs by J. Moravec.

(Fig. 7). Five: two adult males: NMP6V 75653 (sample code IWU 380; MorphoBank pictures: M485674–M485678), NMP6V 75654 (sample code IWU 382) and one juvenile MUSM 32739 (not included in the genetic analyses), all collected at the type locality on 19 May 2014 by Edgar Lehr and Jiří Moravec; one adult female MUSM 32718 (sample code IWU 339; MorphoBank pictures: M485672–M485673) and one juvenile NMP6V 75655 (sample code IWU 340; MorphoBank pictures: M485679–M485680), both collected at the border of the Pui Pui Protected Forest (11.208S, 74.955W; WGS84), 1678 m elevation, Distrito Pichanaqui, Provincia Chanchamayo, Región Junín, Peru, on 12 May 2014 by Edgar Lehr and Jiří Moravec.

###### Diagnosis.

A small gymnophthalmid (SVL 42.1–45.9 mm, n = 4), which can be characterised by the following combination of characters: 1) body slender, slightly depressed, maximum SVL 45.9 mm in males, 42.1 mm in a single female; 2) head relatively short, pointed, about 1.5 times longer than wide; 3) ear opening distinct, moderately recessed; 4) nasals separated by undivided frontonasal; 5) prefrontals, frontal, frontoparietals, parietals, postparietals and interparietal present; 6) parietals slightly longer than wide; 7) supraoculars four, anteriormost fused with anteriormost superciliar; 8) superciliar series complete, consisting of four scales; 9) nasal shield divided above and below or behind the nostril; 10) loreal separated or in contact with second supralabial; 11) supralabials seven; 12) genials in four pairs, first and second pair in contact; 13) collar present, containing 9–11 enlarged scales; 14) dorsals in 33–36 transverse rows, rectangular, nearly twice as long as wide, subimbricate, rugose in adults, slightly keeled in juveniles; 15) ventrals in 22–25 transverse rows, squared to rectangular, smooth, juxtaposed; 16) scales around mid-body 32–34; 17) lateral scales at mid-body reduced in 4–7 lines; 18) limbs pentadactyl, all digits clawed, forelimb reaching anteriorly to third supralabial; 19) subdigital lamellae under Finger IV 14–16, under Toe IV 18–22; 20) femoral pores in males 7–9; 21) four large preanal plate scales; 22) tail about 1.5–1.7 times longer than body (in juveniles); 23) caudals subimbricate, rugose to slightly keeled dorsally in adults, slightly keeled in juveniles, smooth ventrally; 24) lower palpebral disc transparent, undivided; 25) in life, dorsal surface of head, body and limbs light brown with fine dark brown speckling, dorsal surface of tail light brown with a reddish tint or reddish-brown markings; a tan or yellowish brown vertebral stripe bordered laterally by dark brown, vertebral stripe extends on head anteriorly and on tail caudally (inconspicuous in the female); a narrow dirty white to tan dorsolateral line extending on each side from above the tympanum to pelvic region (discontinuous caudally from the level of forelimbs in adults, reaching posterior edge of orbit in some individuals); a narrow dirty white to tan stripe running from above the orbit across parietals and first postparietals up to the neck (connected with the dorsolateral line in some individuals); a narrow white stripe extending from below of orbit to insertion of forelimbs (bordered dorsally by black in juveniles and some adults); minute ocelli-like white spots on flanks (most conspicuous at forearm insertion, absent in some adults); ventrolateral parts of flanks whitish brown; throat and belly creamy white with fine dark grey speckling inside the individual scales (yellowish white with black speckling in juveniles); ventral surfaces of limbs, anal area and tail yellowish white in males and juveniles, white in the female; iris tan with orange tint in males, tan in the female.

###### Description of the holotype.

Body slender; legs moderately long, tail regenerated; head length 22.0% of SVL, head width 14.6% of SVL; snout pointed, moderately long, eye-nose distance 34.7% of HL; neck distinct, collar present; head scales smooth; rostral scale wider than long, slightly higher than adjacent supralabials, in contact with frontonasal, nasals, and first supralabials; frontonasal slightly wider than long, prefrontals present, in wide contact medially; frontal longer than wide, in contact with second and third supraoculars; frontoparietals in contact with third and fourth supraoculars, parietals and interparietal; supraoculars four, none in contact with ciliaries; superciliary series complete, consisting of four shields; anteriormost superciliary fused with anteriormost supraocular, in contact with prefrontal and loreal anteriorly; parietals (left divided) in contact with frontoparietal, fourth supraocular, dorsalmost postocular (separated by small interstitial shield on the left side), one temporal and two postparietals; interparietal longer than wide (divided posteriorly), in contact with three postparietals posteriorly; postparietals six; nasal shield divided above and below the nostril, in contact with first and second supralabial; frenocular triangular, in contact with loreal and second, third and fourth (at one point) supralabial ventrally on the left side and with loreal, nasal (at one point) and second and third supralabial on the right side; palpebral disc oval, translucent, undivided; postoculars three; temporals polygonal, supratympanic temporal one; supralabials seven, fifth below the centre of eye; infralabials six; mental wider than long, in contact with first infralabials; postmental single, in contact with first and second infralabials; genials in four pairs, first and second pair in contact medially, first pair in contact with second and third infralabials, second pair in contact with third and fourth infralabials, third pair in contact with fourth and fifth infralabials, fourth pair in contact with fifth and sixth infralabials; gulars 14; plates in collar 11; dorsal scales homogenous, rectangular, longer than wide, subimbricate, rugose, in 34 transverse rows; dorsals (enlarged scales) across body at fifth transverse ventral scale row 10, at 10^th^ transverse ventral scale row 16, at 15^th^ transverse ventral scale row 16; laterals (smaller lateral scales) at fifth transverse ventral scale row 8–9, at 10^th^ transverse ventral scale row 4–5, at 15^th^ transverse ventral scale row 4–5; ventrals squared to rectangular, juxtaposed, in 23 transverse rows; ventrals across belly at mid-body 10; scales around midbody 32; anterior preanal plate scales two; posterior preanal plate scales four; scales on tail rectangular, subimbricate, slightly keeled dorsally at tail base, smooth and juxtaposed ventrally; subdigital lamellae under Finger IV 14/15 (4/5 distal lamellae single and smooth, remaining lamellae divided in two subconical segments); subdigital lamellae under Toe IV 19/18 (4/4 distal lamellae single and smooth, remaining lamellae divided in two subconical segments); femoral pores 9/7.

###### Measurements of the holotype (in mm).

SVL 45.9; TL (tail regenerated) 38.5; HL 10.1; HW 6.7; HD 5.4; EN 3.5; FLL 11.5; HLL 16.5; AGD 25.0.

###### Colouration of the holotype in life.

(Fig. 6). Head, body, and limbs light brown dorsally with fine dark brown speckling, dorsal surface of tail light brown with reddish brown markings; a tan to yellowish brown vertebral stripe bordered laterally by dark brown, the vertebral stripe is about two dorsal scales wide and extends on the head anteriorly and the tail caudally; a nearly inconspicuous tan dorsolateral line extending on each side from above the tympanum to pelvic region, the line becomes discontinuous and barely visible from the level of forelimbs; a barely visible narrow tan stripe bordered by dark brown ventrally running from above the orbit across parietals and first postparietals and disappearing before reaching the neck; a narrow white stripe bordered by dark brown dorsally extending from below of orbit to insertion of forelimbs; ocelli-like spots on flanks absent; ventrolateral parts of flanks whitish brown; throat and belly creamy white with fine dark grey speckling inside the individual scales; ventral surfaces of limbs, anal area and tail yellowish white; iris tan with an orange tint.

###### Colouration of the holotype in preservative.

General colouration pattern is as described for the holotype in life. The dorsal colouration has a bronze-brown tint, the reddish brown markings on the tail disappeared. Ventral surfaces dirty white with fine dark grey speckling.

###### Hemipenial morphology.

(Fig. 7c; MorphoBank pictures: M485676–M485677). The hemipenes of the paratype NMP6V 75653 were everted during preservation and fixed in alcohol. The completely everted organs measure approximately 5 mm. The hemipenial body has a conical shape with proximal region distinctly thinner than the distal region with lobes. The hemipenial lobes are relatively large, ovoid, distinct from the hemipenial body and do not possess filiform appendages. The flounces on the asulcate side form about 14 discontinuous, but nearly complete, more or less horizontal lines expanding widely on the lateral sides of the distal part of the hemipenial body. There are about seven isolated nearly horizontal flounces on the proximal-central region of the asulcate side. Flounce ornamentation consists of subtle, barely visible denticulation. The sulcus spermaticus begins at the hemipenial base and proceeds in a straight central line towards the lobes. It is edged by lateral fleshy nude areas, which expand in two lateral wings covering the area of lobular division. In that area, the sulcus spermaticus forks into two arms separated by a central fold, which has about eight horizontal ribs. The sulcate arms terminate among lobes and lateral fleshy wings in the apical area of the hemipenis.

###### Variations.

Measurements and scutellation data of the type series are given in Table 5. Colour variation is described in the species diagnosis. In juveniles, the colour pattern is generally brighter than in adults and consists of distinct vertebral and dorsolateral lines and ocelli-like spots on flanks. In the single female, the dorsal colouration is nearly uniformly light brown and the vertebral and dorsolateral lines as well as the ocelli-like spots are poorly developed (Fig. 7.).

**Table 5.** Morphological characters of the type specimens of *Selvasaura brava* sp. n.

Character	MUSM32738 (holotype)	NMP6V75653	NMP6V75654	MUSM32718	MUSM32739	NMP6V75655
Sex	M	M	M	F	Juv	Juv
SVL	45.9	43.9	45.3	42.1	26.8	30.2
TL	–	–	–	–	45.5	44.0
HL	10.1	10.0	10.6	9.8	6.5	6.9
HW	6.7	6.7	7.1	6.6	4.6	4.7
HD	5.4	5.5	5.3	4.8	3.5	3.6
E-N	3.5	3.2	3.6	3.4	2.4	2.4
FLL	11.5	10.5	11.5	10.5	7.5	7.5
HLL	16.5	15.0	16.5	14.5	10.5	10.5
AGD	25.0	22.5	24.4	22.2	13.2	16.5
Supralabials	7/7	7/7	7/7	7/7	7/7	7/7
Scales in collar	11	10	10	11	10	9
Transverse rows of dorsals	34	35	33	34	34	36
Laterals at midbody	6	7	6	6	6	6
Scales around midbody	32	34	34	34	32	33
Transverse rows of ventrals	23	22	24	23	22	25
Ventrals across belly	10	10	10	10	10	10
Preanal plate scales	4	4	4	4	4	4
Lamellae under Finger IV	14/15	15/14	16/15	15/14	14/15	15/16
Lamellae under Toe IV	19/18	19/20	21/21	21/20	21/21	21/22
Femoral pores	9/7	9/8	8/8	–	8/8	–

###### Etymology.

The species epithet *brava* is derived from the Spanish adjective *bravo* (brave, courageous, wild; *brava* the feminine form) and refers to Río Bravo, the largest river in the area of occurrence of the new species, as well as to the fearless nature of the lizard to share shelter with people.

###### Distribution, natural history, and threat status.

**Figure 8.**
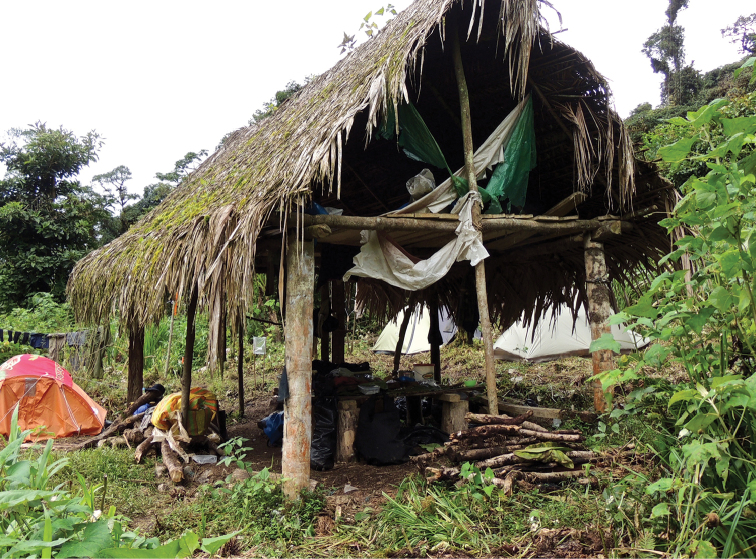
Type locality of *Selvasaura brava* sp. n. The lizards were active during the day basking and foraging in the leaves of the roof and on the shack pillars. They used the leaves on the roof as a refuge to hide in. Photograph by J. Moravec.

*Selvasaura brava* sp. n. is known from two localities lying at the northeastern border of the Pui Pui Protected Forest, ca. 18 km (straight airline distance) NW of the town of Satipo (Fig. 1). Both localities are located in the valley of the tributary of Río Bravo (on opposite banks of the tributary) about 500 m (straight distance) from each other. The valley and its slopes are covered by a primary montane rainforest characterized by 15–20 m high canopy and frequent occurrence of bromeliads, ferns, and epiphytic mosses (see also Lehr and Moravec (2017). All specimens of *S. brava* sp. n. were collected during the day within roofs of provisional camp shacks consisting of dried palm leaves and built by locals on small forest clearings (Fig. 8; MorphoBank picture: M485681). The roofs of the shacks were placed on 1.5–4 m pillars made of tree trunks and stood in an open space fully exposed to sun. The activity of all observed specimens seemed correlated with the intensity of solar radiation. During the sunny hours, the animals emerged from their shelters in the leaf layer, climbed and basked on the roof surface and searched for prey. As agile climbers, the lizards were able to climb up thin vertical tree trunks and jump between the palm leaves. These observations indicate that *S. brava* sp. n. represents an arboreal heliothermic species. Other gymnophthalmid species found at the type locality in sympatry with *S. brava* sp. n. included *Potamites* sp. (not included in the genetic analyses), which inhabited banks of small forest brooks, and *Proctoporus* sp. 4 (sensu this publication, Fig. 3) collected on the ground in the open clearing. With respect to the sparse data available, we suggest classifying *S. brava* as “Data Deficient” according to the IUCN red list criteria.

## Discussion

In this study, we used an unprecedented dataset of nearly all DNA sequences for the cercosaurine lizards available to date to infer a robust phylogeny of the subfamily and to contribute to the knowledge of the biodiversity of the little surveyed montane forests of central Peru. Although more species are being included in the phylogenetic analyses of cercosaurines every year and new phylogenetic hypotheses are being presented, our understanding of the systematics of the subfamily is still far from settled. New genetic data often bring unexpected results that reshuffle the taxonomy of cercosaurines, such as reassignments of species to different genera (Kok 2015; Sánchez-Pacheco et al. 2017b), resurrections of generic names that had once been synonymised (Goicoechea et al. 2012; Chávez et al. 2017), identification of new clades at the genus level (this study; Torres-Carvajal et al. 2016), recognition of cryptic species (Goicoechea et al. 2013), or detection of paraphyletic species or genera (this study; Goicoechea et al. 2012; Torres-Carvajal et al. 2016). Therefore, it is critical to build the phylogenetic trees on extensive taxon sampling, as otherwise many of the above listed issues may go unnoticed.

In concert with previous studies, our results show generally low support for the relationships between the Cercosaurinae genera (Fig. 2). One possibility of the low resolution of the basal nodes is that the group experienced a rapid initial radiation that left few genetic traces that would indicate the actual branching pattern of the cercosaurine evolution. An alternative explanation, and one we find more likely, is that the genetic data currently available for the subfamily (i.e., four mtDNA genes and a single nDNA locus sequenced) are not sufficient for inferring deep-level relationships. We believe that having more nuclear genes sequenced (either by Sanger or next generation sequencing approaches) would improve resolving these basal nodes and shed further light on the monophyly/paraphyly of the questionable genera (see below).

Our results raise many important issues regarding the systematics and taxonomy of the Cercosaurinae that we discuss in detail below.

### Unexpected diversity of genera and species

Recent studies that examined the phylogeny and systematics of the Cercosaurinae on the basis of thorough sampling of taxa (Goicoechea et al. 2012; Torres-Carvajal et al. 2016; Sánchez-Pacheco et al. 2017b) detected previously unknown evolutionary lineages being present in the cercosaurine tree. While most of them could be assigned to currently existing genera (and some have been taxonomically revised since (Goicoechea et al. 2013; Sánchez-Pacheco et al. 2017a)), there were clades whose high levels of genetic divergence and morphological disparity indicated towards the existence of yet unknown clades at the level of genera (Torres-Carvajal et al. 2016). Similarly, our phylogenetic analyses also identified several previously undetected evolutionary lineages.

By formally describing the genus *Selvasaura* we extend the list of currently recognised genera of Cercosaurinae to 16 (Sánchez-Pacheco et al. 2017b). Apart from the formally named genera, there are two clades within cercosaurines that merit genus-level distinction and that are termed here in accordance with previous studies, Unnamed clades 2 and 4. The phylogenetic position of neither of them could be inferred with certainty. Results of both studies in which Unnamed clade 2 was included, this work and Torres-Carvajal et al. (2016), vary in its placement depending on the method of phylogenetic inference. Although all analyses tend to show it close to *Proctoporus* (at least to some groups), lack of support hampers any definitive conclusions regarding its evolutionary origins. We herein provide additional material of ten voucher specimens for Unnamed clade 2 from the PPPF (Table 1), which extends the range of the clade further north compared to previously published localities (Mantaro Valley and Colcabamba-Quintao District, Peru; Torres-Carvajal et al. 2016).

This study is the first to identify a clade that is termed here Unnamed clade 4. As in the case of Unnamed clade 2, the phylogenetic affinities of this clade remain obscure as a result of the low support of deeper level relationships within cercosaurines. Two of the analyses (ML, MrBayes) placed it as sister to *Selvasaura* gen. n. plus *Potamites*, while BEAST placed it as a sister lineage to Unnamed clade 2 (Suppl. material 1: Figs S1–S3). However, none of the topologies was supported. The two specimens that form Unnamed clade 4 represented arboreal lizards caught in cloud forests of southern and central Peru. For the moment, no other data such as morphology, ecology, and natural history are available for the clade as they are being collected and will be published with the formal description of the genus (work in progress).

Besides the newly discovered genera, there are currently 19 unnamed lineages at the level of species in the Cercosaurinae (Fig. 3). They are either morphologically disparate from existing taxa, represent unique genetic lineages or have been delimited as candidate species (this study; Doan et al. 2005; Goicoechea et al. 2012; Torres-Carvajal et al. 2016; Sánchez-Pacheco et al. 2017b) and their systematics and taxonomy should be revised. The presence of so many unidentified species points at the disturbing fact of how little we know about the real diversity of cercosaurine lizards and, perhaps, Neotropical biota in general.

### Paraphyly and polyphyly of genera and species

Some previous studies have already pointed out problems with certain genera not being monophyletic when samples of more species of that genus were included in a phylogenetic analysis. Although many of these issues have been resolved, some still persist or were identified in our study and are to be addressed.

One of the recent examples is paraphyly of the genus *Echinosaura*. As Torres-Carvajal et al. (2016) revealed and we confirmed herein, *E. sulcarostrum* does not cluster with the other species of the genus. The phylogenetic position of this species varies across different phylogenetic analyses. Torres-Carvajal et al. (2016) reconstructed it in their BEAST analysis as a strongly-supported sister lineage to a large clade containing *Anadia*, *Euspondylus* (a name resurrected by Chávez et al. [2017] for the Unnamed clade 1 of Torres-Carvajal et al. [2016]), *Macropholidus*, *Pholidobolus*, *Petracola*, *Cercosaura*, *Selvasaura* (termed Unnamed clade 3 in their paper), *Potamites*, *Proctoporus*, and Unnamed clade 2; their ML analysis recovered it as sister to *Proctoporus xestus*. Subsequently, the genetic analysis of Sánchez-Pacheco et al. (2017b) recovered it as sister to a clade of *Oreosaurus*, *Potamites*, *Petracola*, *Cercosaura*, and *Proctoporus*, although with a limited taxon sampling. Finally, our analyses yielded it as sister to *Euspondylus*, although the topology was supported only in the MrBayes analysis. Regardless, none of the analyses found it close to the other *Echinosaura* species including *E. horrida*, the type species of the genus. As a result, its taxonomy, as well as proper phylogenetic placement, remain to be revised.

Another possible case of paraphyly is the genus *Oreosaurus* that was recently resurrected by Sánchez-Pacheco et al. (2017b). While the authors found the genus monophyletic, our analyses yielded no support for the clade as a whole with *O. serranus* placed separately from the other species. It was found sister to the remaining species only in our BEAST analysis, but support for the basal node was very low (pp = 0.3) indicating that 70% of the posterior trees actually had a different topology. Such a discrepancy between our and Sánchez-Pacheco et al. (2017b) results may stem from the difference in the analytical approaches undertaken. All our phylogenetic analyses were model-based, i.e., we assumed the sequences to evolve under evolutionary models that take into account the variation of substitution rates among sites in the alignments and the possibility of recurrent mutations at one site. On the contrary, Sánchez-Pacheco et al. (2017b) performed a maximum parsimony analysis that reconstructs the phylogeny based on the smallest number of evolutionary events necessary to explain the sequence data. Both methods may under some circumstances result in different topologies, especially when analysing relatively distant taxa where long branch attraction can occur (Felsenstein 1978; Alfaro et al. 2003). This may be the case here considering that we deal with a group whose origin has been estimated to have taken place in the early Tertiary (Zheng and Wiens 2016). Regarding the morphology, Sánchez-Pacheco et al. (2017b) found the genus *Oreosaurus* being clearly different from *Andinosaura* and *Riama* by lacking a narrow band of differentiated granular lateral scales. On the other hand, Sánchez-Pacheco et al. (2017a) show that *Oreosaurus serranus* can be distinguished morphologically from all other *Oreosaurus* species by having only one pair of genial scales. Therefore, taking into account the above-mentioned discrepancies in the results of molecular analyses, and the morphological distinctiveness of *O. serranus* from other *Oreosaurus* species, the character of nucleotide divergence between *O. serranus* and the other *Oreosaurus* species should be examined in detail in order to trace the inconsistency in the phylogenetic reconstructions.

At the species level, recent taxonomic advances made possible by the tremendous effort of many authors are making considerable progress in stabilising the taxonomy of cercosaurines (e.g., Goicoechea et al. 2013; Torres-Carvajal et al. 2014; Venegas et al. 2016; Sánchez-Pacheco et al. 2017a; among many others), yet non-monophyletic species are still present in the phylogeny. For example, the seven samples of *Potamites ecpleopus* used in this study form two groups, one is distributed in eastern Ecuador and the other in northeastern and southern Peru. Paraphyly of this species was already noted by Torres-Carvajal et al. (2016). Because the type locality of the species lies approximately in the centroid of the crescent delineated by the sampled localities, it is impossible to assign the species name to either of the groups with certainty until a comprehensive revision with specimens from the type locality is undertaken. Another example are two *Cercosaura* species, *C. parkeri* and *C. schreibersii*, which are paraphyletic with respect to each other as also noted by Sturaro et al. (2017). *Cercosaura parkeri* was originally described as a subspecies of *C. schreibersii* (Ruibal 1952) and elevated to species status by Tedesco and Cei (1999), but a further taxonomic revision of the species complex is apparently needed to resolve the remaining issues.

### Species groups in *Proctoporus*

Yet another genus in which between-species relationships have proven difficult to infer is *Proctoporus* (Doan 2003; Doan et al. 2005; Torres-Carvajal et al. 2016), and this study supports this notion (but see Goicoechea et al. 2012). These semi-fossorial lizards inhabit primarily montane forests between 1000 m and 4000 m of altitude from central Peru to central Bolivia (Uzzell 1970; Goicoechea et al. 2012). Although certain species groups have traditionally been identified, mutual relationships between them and to other cercosaurine genera remain poorly resolved. Given the amount of cryptic species present within the genus (8 undescribed or candidate species; Fig. 3) indicating that future taxonomic revisions are to be expected, we herein propose the following terminology of the species groups in order to facilitate addressing this issue in future studies. The species groups are: (1) *Proctoporus pachyurus* group that contains *P. chasqui*, *P. oreades*, *P. pachyurus*, *P. rahmi*, *P. spinalis*, *P. sucullucu*, and three yet undescribed species (labelled *P.* sp., *P.* sp. 1, *P.* sp. 5); (2) *Proctoporus lacertus* group that consists of four recently described or resurrected species (*P. carabaya*, *P. iridescens*, *P. kiziriani*, *P. lacertus*), which were formerly considered part *P. bolivianus* (Goicoechea et al. 2012; 2013); (3) *Proctoporus bolivianus* group of two species, *P. bolivianus* and a confirmed candidate species (labelled *P.* Ca1 following Goicoechea et al. [2012, 2013]); (4) *Proctoporus guentheri* group, which contains the highest proportion of undescribed species (four, labelled *P.* sp. 2, *P.* sp. 3, *P.* sp. 4, *P.* Ca2 following Goicoechea et al. [2012, 2013]) besides three described species (*P. guentheri*, *P. laudahnae*, *P. unsaacae*); and (5) a clade of a single species, *P. xestus*. Most recent phylogenetic reconstructions of the genus were based on identical sampling of loci (three mitochondrial [12S, 16S, ND4] and one nuclear marker [cmos]) and our study has added sequences of one additional mtDNA marker (cytb) for only two species. The congruence of results obtained across studies and showing little support for the basal nodes is thus not surprising. We believe that getting a better resolution of the relationships between the *Proctoporus* groups is a matter of better sampling of loci and that more nuclear markers sequenced would shed more light on this subject.

### Phylogenetic placement of generic type species

The above problems with non-monophyletic genera raise an important nomenclatural issue regarding the application of generic names. Generic names apply to clades that contain the type species of the genus. In cases when genera are formed by more unrelated evolutionary lineages (e.g., *Echinosaura*) inferring the phylogenetic position of the type species is the only way to determine which of the lineages will bear the genus name; the other has to be renamed. In cercosaurines, most type species have been sequenced and placed in the phylogenetic context of the subfamily (for type species see Uetz et al. 2018), and this study provides an important addition to it.

For the first time, we sequenced the type species of the genus *Anadia* (*A. ocellata*). The sample clusters with other congeneric species in the dataset and thus fixes the name *Anadia* to this clade. Most species of *Anadia* have not been sequenced yet (Fig. 2), and it cannot be ruled out that once they are included in phylogenetic analyses, more cases of paraphyly will be detected. Similar situations have occurred in *Anadia* before when *A. mcdiarmidi* was found not to cluster with other species of the genus (Torres-Carvajal et al. 2016) and was subsequently reassigned to the genus *Oreosaurus* (Sánchez-Pacheco et al. 2017b).

Currently, the only cercosaurine genera with type species missing from the phylogenetic trees presented here are *Euspondylus* (type species *E. maculatus*) and *Oreosaurus* (type species *O. luctuosus*). Obtaining DNA sequences of the latter is particularly desired, as including its samples in phylogenetic analyses should help resolving the issue with the potential paraphyly in *Oreosaurus* (see above).

### Montane forests of Peru

Montane forests (región yunga or selva alta) are found in the eastern Andes roughly between 800 and 3500 m a.s.l. (Perú, Ministerio del Ambiente 2015) and are known for their high biodiversity and an increasing endemism with increasing elevation (Young and León 2000). Yet, montane forests are among the least studied and least understood ecosystems (Ledo et al. 2012). In a recent vegetation map of Peru, 12 different types of montane forests were recognized within the región yunga, covering in total 9.58% of the national territory (Perú, Ministerio del Ambiente 2015). Its dense vegetation and steep slopes make the herpetofauna of montane forests relatively difficult to survey, and the canopy herpetofauna is probably the least known. However, exciting discoveries often happen by accident (e.g., arboreal species found in bromeliads on a fallen tree, Duellman et al. 2004).

All specimens of *Selvasaura brava* sp. n. were found in secondary forests, hiding in the roofs of simple wooden shacks where specimens could be easily seen and caught, whereas not a single specimen was found in primary forests. Our discovery of the new cercosaurine clade of arboreal lizards (Unnamed clade 4) together with a recent description of a new arboreal *Euspondylus* from central Peru (Chávez et al. 2017) indicate that arboreal species of cercosaurines may be much more diverse than previously thought and further research will be necessary to fully understand their diversity and ecology.

Some members of several cercosaurine genera (*Anadia*, *Euspondylus*, *Selvasaura*, Unnamed clade 4) are adapted to life in the above-ground vegetation (Oftedal 1974; Chávez et al. 2017; this study) and certain species of some other genera also show tendency to arboreality (e.g., *Cercosaura*; Vitt et al. 2003). Repeated convergent adaptation to arboreality in Neotropical lizards has been well documented and studied in anoles (Losos 1990; 1992; Kolbe et al. 2011). If such is the case for Cercosaurinae can only be answered when we have a better-resolved phylogeny. Furthermore, no such studies have been conducted to compare different arboreal lifestyles and arboreal locomotion (see Fischer et al. (2010) for definitions) in Cercosaurinae, nor have different ecomorphs, and their adaptations to their arboreal niches been described. We note the small size of *Selvasaura brava* (SVL = 42.1–45.9 mm, n = 4) and the relatively short front and hind limbs, yet detailed observations of their locomotory behaviour in nature are missing.

## Supplementary Material

XML Treatment for Selvasaura

XML Treatment for Selvasaura brava

## Supplementary materials

Supplementary material 1 Supplementary phylogenetic trees and table of analysed samples Data type: molecular data This is an open access article distributed under the terms of the Creative Commons Attribution License (CC BY 4.0), which permits unrestricted use, distribution, and reproduction in any medium, provided the original author and source are credited.Jiří Moravec, Jiří Šmíd, Jan Štundl, Edgar Lehr
