# Supplementary material for: Systematics of Neotropical microteiid lizards (Gymnophthalmidae, Cercosaurinae), with the description of a new genus and species from the Andean montane forests
Source: Zookeys. 2018 Jul 16;(774):105–39. doi: 10.3897/zookeys.774.25332 (PMC6056569; doi:10.3897/zookeys.774.25332)
Supplement: Supplementary material 1 — Supplementary phylogenetic trees and table of analysed samples [file zookeys-774-105-s001.pdf]

ZooKeys

Systematics of Neotropical microteiid lizards (Gymnophthalmidae, Cercosaurinae), with the description of a new genus and species from the Andean montane forests

Jiří Moravec, Jiří Šmíd, Jan Štundl, Edgar Lehr

SUPPLEMENTARY MATERIAL

Contents:

Figure S1. A complete tree of Cercosaurinae and outgroup taxa resulting from the ML analysis.

Figure S2. A 50% majority-rule consensus tree of Cercosaurinae and outgroup taxa resulting from the MrBayes analysis.

Figure S3. An MCC tree of Cercosaurinae and outgroup taxa resulting from the BEAST analysis.

Table S1. Samples of the Cercosaurinae and outgroup species used for the phylogenetic analyses and their respective GenBank accession numbers. Sample codes are those shown in tree figures. Voucher numbers correspond to those given in the original references.

References to Table S1.

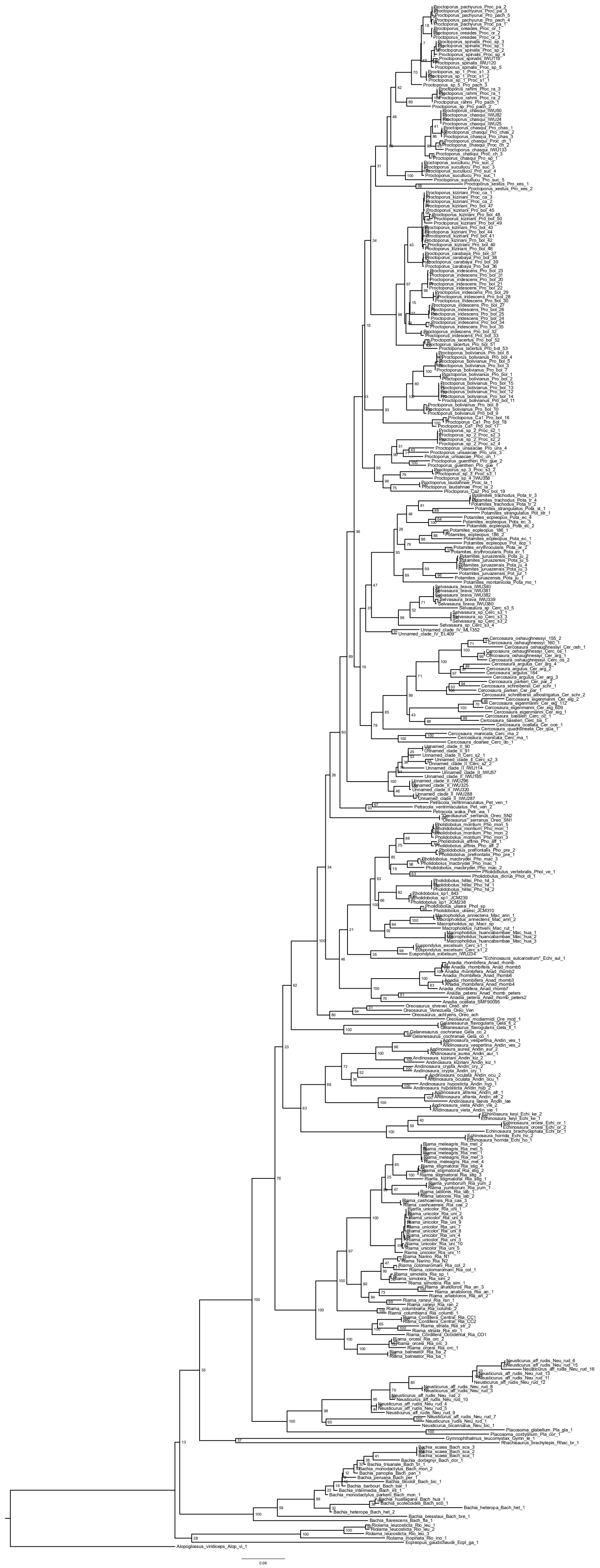

Figure S1. A complete tree of Cercosaurinae and outgroup taxa resulting from the ML analysis.

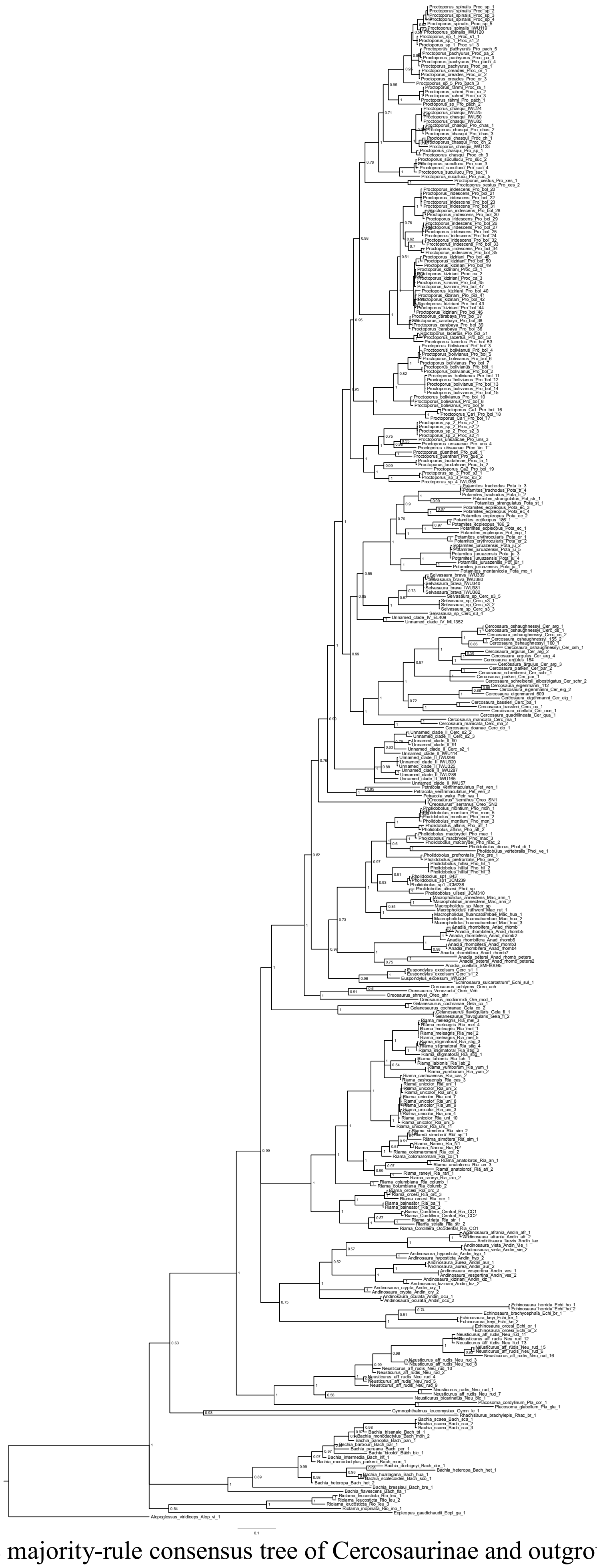

Figure S2. A 50% majority-rule consensus tree of Cercosaurinae and outgroup taxa resulting from the MrBayes analysis.

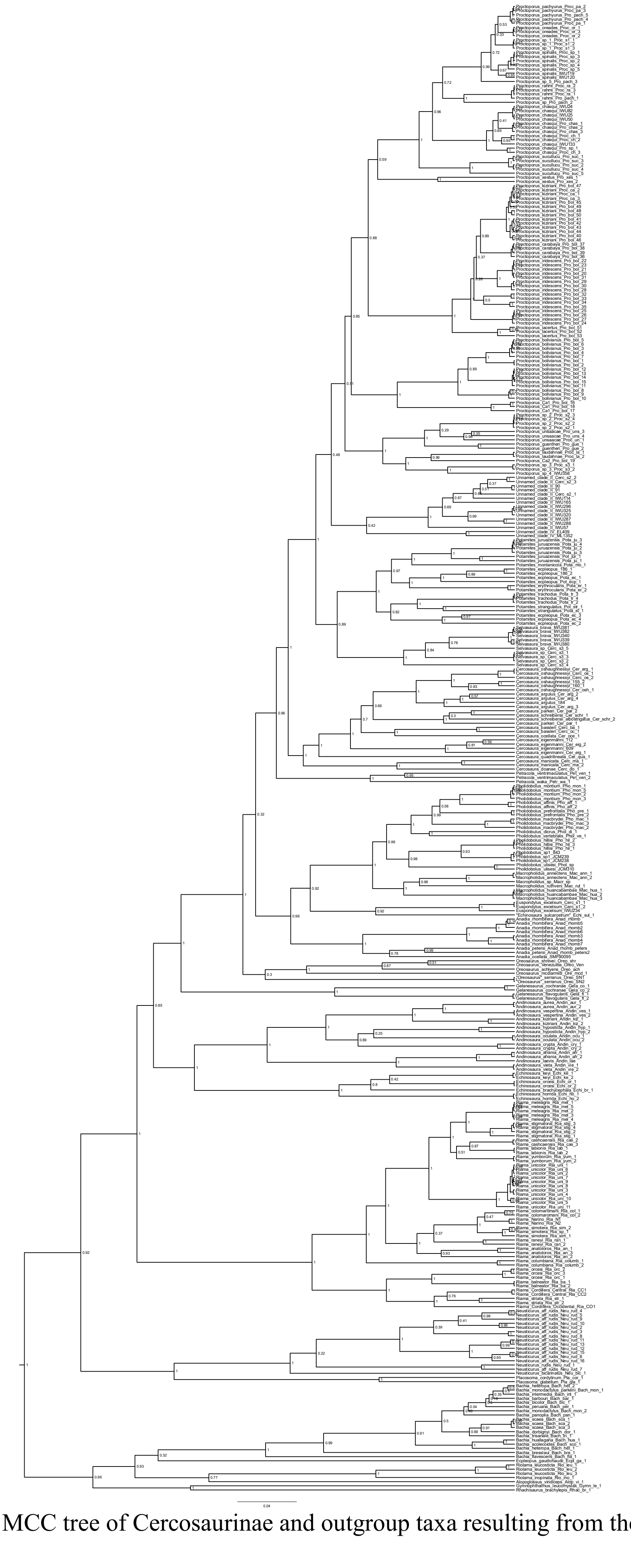

Figure S3. An MCC tree of Cercosaurinae and outgroup taxa resulting from the BEAST analysis.

Table S1. Samples of the Cercosaurinae and outgroup species used for the phylogenetic analyses and their respective GenBank accession numbers. Sample codes are those shown in tree figures. Voucher numbers correspond to those given in the original references.

Genus	Species	Sample code	Voucher	GenBank Accession					Source
				12S	16S	cytb	ND4	c-mos	
Anadia	*A. ocellata*	SMF90095	SMF90095	MH579588	MH579625	MH579659			This study
Anadia	*A. petersi*	Anad_rhomb_peters	QCAZ 5068	KU902127	KU902208		KU902283	KU902048	Torres-Carvajal et al. 2016
Anadia	*A. petersi*	Anad_rhomb_peters2	QCAZ 10087	KU902130	KU902211		KU902286	KU902050	Torres-Carvajal et al. 2016
Anadia	*A. rhombifera*	Anad_rhomb	QCAZ 5782	KU902128	KU902209		KU902284		Torres-Carvajal et al. 2016
Anadia	*A. rhombifera*	Anad_rhomb2	QCAZ 6873	KU902129	KU902210		KU902285	KU902049	Torres-Carvajal et al. 2016
Anadia	*A. rhombifera*	Anad_rhomb3	QCAZ 10537	KU902131	KU902212		KU902287		Torres-Carvajal et al. 2016
Anadia	*A. rhombifera*	Anad_rhomb4	QCAZ 10556	KU902132	KU902213		KU902288	KU902051	Torres-Carvajal et al. 2016
Anadia	*A. rhombifera*	Anad_rhomb5	QCAZ 11061	KU902133	KU902214		KU902289	KU902052	Torres-Carvajal et al. 2016
Anadia	*A. rhombifera*	Anad_rhomb6	QCAZ 11510	KU902134	KU902215		KU902290		Torres-Carvajal et al. 2016
Anadia	*A. rhombifera*	Anad_rhomb7	QCAZ 11862	KU902135	KU902216		KU902291	KU902053	Torres-Carvajal et al. 2016
Andinosaura	*A. afrania*	Andin_afr_1	RH	KY670680	KY681098				Sanchez-Pacheco et al. 2017
Andinosaura	*A. afrania*	Andin_afr_2	RM	KY670681	KY681099				Sanchez-Pacheco et al. 2017
Andinosaura	*A. aurea*	Andin_aur_1	QCAZ9649	KY670682	KY681100		KY710831	KY670647	Sanchez-Pacheco et al. 2017
Andinosaura	*A. aurea*	Andin_aur_2	QCAZ9650	KY670683	KY681101		KY710832	KY670648	Sanchez-Pacheco et al. 2017
Andinosaura	*A. crypta*	Andin_cry_1	QCAZ10455	KY670684	KY681102		KY710833	KY670649	Sanchez-Pacheco et al. 2017
Andinosaura	*A. crypta*	Andin_cry_2	QCAZ6154	KY670685	KY681103		KY710834	KY670650	Sanchez-Pacheco et al. 2017
Andinosaura	*A. hyposticta*	Andin_hyp_1	PSO-CZ85	KY670686	KY681104				Sanchez-Pacheco et al. 2017
Andinosaura	*A. hyposticta*	Andin_hyp_2	DHMECN1360	KY670687	KY681105			KY670651	Sanchez-Pacheco et al. 2017
Andinosaura	*A. kiziriani*	Andin_kiz_2	QCAZ9667	KY670689	KY681107		KY710836	KY670653	Sanchez-Pacheco et al. 2017
Andinosaura	*A. kiziriani*	Andin_kiz_1	QCAZ9607	KY670688	KY681106		KY710835	KY670652	Sanchez-Pacheco et al. 2017
Andinosaura	*A. laevis*	Andin_lae	WB1330	KY670690	KY681108		KY799165	KY670654	Sanchez-Pacheco et al. 2017
Andinosaura	*A. oculata*	Andin_ocu_1	QCAZ10410	KY670691	KY681109		KY710837	KY670655	Sanchez-Pacheco et al. 2017
Andinosaura	*A. oculata*	Andin_ocu_2	QCAZ5474	KY670692	KY681110		KY710838	KY670656	Sanchez-Pacheco et al. 2017
Andinosaura	*A. vespertina*	Andin_ves_1	QCAZ10286	KY670693	KY681111		KY710839	KY670657	Sanchez-Pacheco et al. 2017
Andinosaura	*A. vespertina*	Andin_ves_2	QCAZ10306	KY670694	KY681112		KY710840	KY670658	Sanchez-Pacheco et al. 2017
Andinosaura	*A. vieta*	Andin_vie_1	QCAZ10456	KY670695	KY681113		KY710841	KY670659	Sanchez-Pacheco et al. 2017
Andinosaura	*A. vieta*	Andin_vie_2	QCAZ5287	KY670696	KY681114		KY710842	KY670660	Sanchez-Pacheco et al. 2017
Cercosaura	*C. argulus*	Cer_arg_2	QCAZ 4888	KP874738	KP874790		KP874900	KP874842	Torres-Carvajal et al. 2016
Cercosaura	*C. argulus*	Cer_arg_3	MPEG21580	KY555149	KY555221		KY555431	KY555359	Sturaro et al. 2017
Cercosaura	*C. argulus*	Cer_arg_4	MPEG21540	KY555148	KY555220		KY555362	KY555360	Sturaro et al. 2017

Genus	Species	Sample code	Voucher	GenBank Accession					Source
				12S	16S	cytb	ND4	c-mos	
Cercosaura	*C. argulus*	184	NMP6V 72184	MH579589	MH579626	MH579660		MH579686	This study
Cercosaura	*C. bassleri*	Cerc_ba_1	CORBIDI 15187	KU902136	KU902217		KU902292	KU902054	Torres-Carvajal et al. 2016
Cercosaura	*C. bassleri*	Cerc_oc_1	CORBIDI 11218	KP874744	KP874796		KP874906	KP874848	Torres-Carvajal et al. 2016
Cercosaura	*C. doanae*	Cerc_do_1	CORBIDI 650	KP874773	KP874825		KP874935	KP874875	Torres-Carvajal et al. 2016
Cercosaura	*C. eigenmanni*	112	NMP6V 73112	MH579591	MH579628	MH579661			This study
Cercosaura	*C. eigenmanni*	609	NMP6V 72609	MH579590	MH579627				This study
Cercosaura	*C. eigenmanni*	Cer_eig_1	MRT 976979	AF420690	AF420728		AF420895	AF420828	Pellegrino et al. 2001
Cercosaura	*C. eigenmanni*	Cer_eig_2	MPEG21550	KY555150	KY555222		KY555361	KY555358	Sturaro et al. 2017
Cercosaura	*C. manicata*	Cerc_ma_1	CORBIDI 8837	KP874745	KP874797		KP874907	KP874849	Torres-Carvajal et al. 2016
Cercosaura	*C. manicata*	Cerc_ma_2	QCAZ 5793	KP874746	KP874798		KP874908	KP874850	Torres-Carvajal et al. 2016
Cercosaura	*C. ocellata*	Cer_oce_1	MRT 977406	AF420677	AF420731		AF420883	AF420834	Pellegrino et al. 2001
Cercosaura	*C. oshaughnessyi*	Cer_arg_1	LSUMZ H12591	AF420698	AF420751		AF420896	AF420838	Pellegrino et al. 2001<br>Torres-Carvajal et al. 2016
Cercosaura	*C. oshaughnessyi*	Cer_osh_1	LSUMZ H13584	AF420696	AF420750		AF420893	AF420852	Pellegrino et al. 2001
Cercosaura	*C. oshaughnessyi*	Cerc_os_1	LSUMZ H12591	AF420698	AF420751		AF420896	AF420838	Pellegrino et al. 2001<br>Torres-Carvajal et al. 2016
Cercosaura	*C. oshaughnessyi*	Cerc_os_2	QCAZ 4623	KP874748	KP874800		KP874910	KP874852	Torres-Carvajal et al. 2016
Cercosaura	*C. oshaughnessyi*	155/2	NMP6V 71155/2	MH579592					This study
Cercosaura	*C. oshaughnessyi*	160/1	NMP6V 71160/1	MH579593	MH579629	MH579662		MH579687	This study
Cercosaura	*C. parkeri*	Cer_par_1	LG1560	KY555219	KY555290		KY555378	KY555291	Sturaro et al. 2017
Cercosaura	*C. parkeri*	Cer_par_2	LG1553	KY555218	KY555289		KY555363	KY555292	Sturaro et al. 2017
Cercosaura	*C. quadrilineata*	Cer_qua_1	LG 936	AF420672	AF420717		AF420880	AF420830	Pellegrino et al. 2001
Cercosaura	*C. schreibersii*	Cer_schr_1	LG 927	AF420686	AF420749		AF420911	AF420817	Goicoechea et al. 2012
Cercosaura	*C. s. albostrigatus*	Cer_schr_2	LG 1168	AF420658	AF420729		AF420882	AF420856	Pellegrino et al. 2001
Echinosaura	*E. brachycephala*	Echi_br_1	QCAZ 10824	KU902137	KU902218		KU902293	KU902055	Torres-Carvajal et al. 2016
Echinosaura	*E. horrida*	Echi_ho_1	QCAZ 6666	KU902138	KU902219		KU902294	KU902056	Torres-Carvajal et al. 2016
Echinosaura	*E. horrida*	Echi_ho_2	QCAZ 8788	KU902139	KU902220		KU902295	KU902057	Torres-Carvajal et al. 2016
Echinosaura	*E. keyi*	Echi_ke_1	QCAZ 8074	KU902140	KU902221		KU902296	KU902058	Torres-Carvajal et al. 2016
Echinosaura	*E. keyi*	Echi_ke_2	QCAZ 12485	KU902141	KU902222		KU902297	KU902059	Torres-Carvajal et al. 2016
Echinosaura	*E. orcesi*	Echi_or_1	QCAZ 6299	KU902142	KU902223		KU902298	KU902060	Torres-Carvajal et al. 2016
Echinosaura	*E. orcesi*	Echi_or_2	QCAZ 10022	KU902143	KU902224		KU902299	KU902061	Torres-Carvajal et al. 2016
"Echinosaura"	*E. sulcarostrum*	Echi_sul_1	ROM 22892	AF206584	AF206584	AF206528			Fu 2000<br>redetermined by Kok 2015
Euspondylus	*E. excelsum*	Cerc_s1_1	CORBIDI 14965	KU902198	KU902273		KU902354	KU902117	Torres-Carvajal et al. 2016
Euspondylus	*E. excelsum*	Cerc_s1_2	CORBIDI 15573	KU902199	KU902274		KU902355	KU902118	Torres-Carvajal et al. 2016

Genus	Species	Sample code	Voucher	GenBank Accession					Source
				12S	16S	cytb	ND4	c-mos	
Euspondylus	*E. excelsum*	IWU234	IWU 234	MH579594	MH579630			MH579688	This study
Gelanesaurus	*G. cochranae*	Gela_co_1	QCAZ 5587	KU902144	KU902225		KU902300	KU902062	Torres-Carvajal et al. 2016
Gelanesaurus	*G. cochranae*	Gela_co_2	QCAZ 6120	KU902145	KU902226		KU902301	KU902063	Torres-Carvajal et al. 2016
Gelanesaurus	*G. flavogularis*	Gela_fl_1	QCAZ 4611	KU902146	KU902227		KU902302	KU902064	Torres-Carvajal et al. 2016
Gelanesaurus	*G. flavogularis*	Gela_fl_2	QCAZ 6943	KU902147	KU902228		KU902303	KU902065	Torres-Carvajal et al. 2016
Macropholidus	*M. annectens*	Mac_ann_1	QCAZ 11120	KC894341	KC894355		KC894369		Torres-Carvajal and Mafla-Endara 2013
Macropholidus	*M. annectens*	Mac_ann_2	QCAZ 11121	KC894342	KC894356		KC894370	KP874877	Torres-Carvajal and Mafla-Endara 2013
Macropholidus	*M. huancabambae*	Mac_hua_1	CORBIDI 10492	KC894343	KC894357		KC894371		Torres-Carvajal and Mafla-Endara 2013
Macropholidus	*M. huancabambae*	Mac_hua_2	CORBIDI 10493	KC894344	KC894358		KC894372	KP874879	Torres-Carvajal and Mafla-Endara 2013
Macropholidus	*M. huancabambae*	Mac_hua_3	CORBIDI 10496	KC894345	KC894359		KC894373		Torres-Carvajal and Mafla-Endara 2013
Macropholidus	*M. ruthveni*	Mac_rut_1	CORBIDI 4281	KC894354	KC894368		KC894382		Torres-Carvajal and Mafla-Endara 2013
Macropholidus	*M.* sp.	Macr_sp	CORBIDI 12932	KP874774	KP874826		KP874936	KP874881	Torres-Carvajal et al. 2016
Neusticurus	*N.* aff. *rudis*	Neu_rud_10	ROM42644		JQ742249				Kok et al. 2012
Neusticurus	*N.* aff. *rudis*	Neu_rud_11	IRSNB18109		JQ742251				Kok et al. 2012
Neusticurus	*N.* aff. *rudis*	Neu_rud_12	IRSNB18110		JQ742252				Kok et al. 2012
Neusticurus	*N.* aff. *rudis*	Neu_rud_13	IRSNB18111		JQ742253				Kok et al. 2012
Neusticurus	*N.* aff. *rudis*	Neu_rud_15	IRSNB18150		JQ742240				Kok et al. 2012
Neusticurus	*N.* aff. *rudis*	Neu_rud_16	IRSNB18151		JQ742241				Kok et al. 2012
Neusticurus	*N.* aff. *rudis*	Neu_rud_2	IRSNB17344		JQ742242				Kok et al. 2012
Neusticurus	*N.* aff. *rudis*	Neu_rud_3	IRSNB17345		JQ742243				Kok et al. 2012
Neusticurus	*N.* aff. *rudis*	Neu_rud_4	IRSNB18146		JQ742244				Kok et al. 2012
Neusticurus	*N.* aff. *rudis*	Neu_rud_5	IRSNB18147		JQ742245				Kok et al. 2012
Neusticurus	*N.* aff. *rudis*	Neu_rud_6	PK2058V		JQ742246				Kok et al. 2012
Neusticurus	*N.* aff. *rudis*	Neu_rud_7	IRSNB18149		JQ742250				Kok et al. 2012
Neusticurus	*N.* aff. *rudis*	Neu_rud_8	ROM20514		JQ742247				Kok et al. 2012
Neusticurus	*N.* aff. *rudis*	Neu_rud_9	ROM39498		JQ742248				Kok et al. 2012
Neusticurus	*N. bicarinatus*	Neu_bic_1	MRT 968462	AF420671	AF420708			AF420816	Goicoechea et al. 2012
Neusticurus	*N. rudis*	Neu_rud_1	MRT 926008	AF420689	AF420709		AF420905		Goicoechea et al. 2012
Oreosaurus	*O.* "Venezuela"	Oreo_Ven	GAR5962	KY670701	KY681119		KY799161		Sanchez-Pacheco et al. 2017
Oreosaurus	*O. achlyens*	Oreo_ach	ENS11010	KY670697	KY681115		KY799160		Sanchez-Pacheco et al. 2017
Oreosaurus	*O. mcdiarmidi*	Ore_mcd_1	IRSNB 2674		JQ742263		KP283392	KP283385	Kok 2015
Oreosaurus	*O. shrevei*	Oreo_shr	UWIZM2011.7	KY670700	KY681118			KY670663	Sanchez-Pacheco et al. 2017
"Oreosaurus"	*O. serranus*	Oreo_SN2	JJS548	KY670699	KY681117		KY799164	KY670662	Sanchez-Pacheco et al. 2017
"Oreosaurus"	*O. serranus*	Oreo_SN1	JJS543	KY670698	KY681116		KY799163	KY670661	Sanchez-Pacheco et al. 2017

Genus	Species	Sample code	Voucher	GenBank Accession					Source
				12S	16S	cytb	ND4	c-mos	
Petracola	*P. ventrimaculatus*	Pet_ven_1	KU219838	AY507863	AY507883		AY507894	AY507910	Doan et al. 2005
Petracola	*P. ventrimaculatus*	Pet_ven_2	CORBIDI 9235	KJ948193	KJ948144		KJ948145	KJ948220	Aguirre-Penafiel et al. 2014
Petracola	*P. waka*	Petr_wa_1	KU212687	AY507864	AY507876			AY507903	Castoe et al. 2004
Pholidobolus	*P. affinis*	Pho_aff_1	QCAZ 9641	KC894348	KC894362		KC894376	KP874883	Torres-Carvajal and Mafla-Endara 2013
Pholidobolus	*P. affinis*	Pho_aff_2	QCAZ 9900	KC894349	KC894363		KC894377		Torres-Carvajal and Mafla-Endara 2013
Pholidobolus	*P. dicrus*	Phol_di_1	QCAZ 5304	KP874776	KP874828		KP874938	KP874885	Torres-Carvajal et al. 2016
Pholidobolus	*P. hillisi*	Pho_hil_1		KP090167	KP090170		KP090173		Torres-Carvajal et al. 2014
Pholidobolus	*P. hillisi*	Pho_hil_2	QCAZ 5000	KP090168	KP090171		KP090174	KP874888	Torres-Carvajal et al. 2014
Pholidobolus	*P. hillisi*	Pho_hil_3		KP090169	KP090172		KP090175		Torres-Carvajal et al. 2014
Pholidobolus	*P. macbrydei*	Pho_mac_1	KU218406	AY507848	AY507867		AY507886	AY507896	Doan et al. 2005
Pholidobolus	*P. macbrydei*	Pho_mac_2	QCAZ 9914	KC894352	KC894366		KC894380		Torres-Carvajal and Mafla-Endara 2013
Pholidobolus	*P. macbrydei*	Pho_mac_3	QCAZ 9932	KC894353	KC894367		KC894381	KP874889	Torres-Carvajal and Mafla-Endara 2013
Pholidobolus	*P. montium*	Pho_mon_1		AF420701	AF420756		AF420884	AF420820	Goicoechea et al. 2012
Pholidobolus	*P. montium*	Pho_mon_2	QCAZ 4051	KC894346	KC894360		KC894374	KP874890	Torres-Carvajal and Mafla-Endara 2013
Pholidobolus	*P. montium*	Pho_mon_3	QCAZ 9044	KC894347	KC894361		KC894375		Torres-Carvajal and Mafla-Endara 2013
Pholidobolus	*P. montium*	Pho_mon_5	QCAZ8056	KJ948194	KJ948143		KJ948146	KJ948221	Aguirre-Penafiel et al. 2014
Pholidobolus	*P. prefrontalis*	Pho_pre_1	QCAZ 9908	KC894350	KC894364		KC894378		Torres-Carvajal and Mafla-Endara 2013
Pholidobolus	*P. prefrontalis*	Pho_pre_2	QCAZ 9951	KC894351	KC894365		KC894379		Torres-Carvajal and Mafla-Endara 2013
Pholidobolus	*P.* sp.1	843	MUSM 31843	MH579597	MH579633	MH579665		MH579691	This study
Pholidobolus	*P.* sp.1	JCM238	JCM 238	MH579595	MH579631	MH579663		MH579689	This study
Pholidobolus	*P.* sp.1	JCM239	JCM 239	MH579596	MH579632	MH579664		MH579690	This study
Pholidobolus	*P. ulisesi*	JCM310	JCM 310	MH579598	MH579634	MH579666		MH579692	This study
Pholidobolus	*P. ulisesi*	Phol_sp	CORBIDI 12737	KP874788	KP874840		KP874949	KP874898	Torres-Carvajal et al. 2016
Pholidobolus	*P. vertebralis*	Phol_ve_1	QCAZ 8688	KP874780	KP874832		KP874942	KP874892	Torres-Carvajal et al. 2016
Placosoma	*P. cordylinum*	Pla_cor_1	LG 1006	AF420673	AF420734		AF420879	AF420823	Goicoechea et al. 2012
Placosoma	*P. glabellum*	Pla_gla_1		AF420674	AF420742		AF420907	AF420833	Goicoechea et al. 2012
Potamites	*P. ecpleopus*	186/1	NMP6V 73186/1	MH579599	MH579635				This study
Potamites	*P. ecpleopus*	186/2	NMP6V 73186/2	MH579600	MH579636				This study
Potamites	*P. ecpleopus*	Pot_ecp_1	MRT 0472	AF420656	AF420748		AF420890	AF420829	Goicoechea et al. 2012
Potamites	*P. ecpleopus*	Pota_ec_1	CORBIDI 14382	KU902148	KU902229		KU902304	KU902066	Torres-Carvajal et al. 2016
Potamites	*P. ecpleopus*	Pota_ec_2	QCAZ 4699	KU902149	KU902230		KU902305	KU902067	Torres-Carvajal et al. 2016
Potamites	*P. ecpleopus*	Pota_ec_3	QCAZ 5208	KU902150	KU902231		KU902306	KU902068	Torres-Carvajal et al. 2016
Potamites	*P. ecpleopus*	Pota_ec_4	QCAZ 10071	KU902151	KU902232		KU902307	KU902069	Torres-Carvajal et al. 2016
Potamites	*P. erythrocularis*	Pota_er_1	CORBIDI 15153	KU902152	KU902233		KU902308	KU902070	Torres-Carvajal et al. 2016

Genus	Species	Sample code	Voucher	GenBank Accession					Source
				12S	16S	cytb	ND4	c-mos	
Potamites	*P. erythrocularis*	Pota_er_2	MHNC 10946	KU902153	KU902234		KU902309	KU902071	Torres-Carvajal et al. 2016
Potamites	*P. juruazensis*	Pot_jur_1					AF420878	AF420857	Pellegrino et al. 2001
Potamites	*P. juruazensis*	Pota_ju_1	CORBIDI 9951	KU902154	KU902235		KU902310	KU902072	Torres-Carvajal et al. 2016
Potamites	*P. juruazensis*	Pota_ju_2	CORBIDI 15504	KU902155	KU902237		KU902312	KU902074	Torres-Carvajal et al. 2016
Potamites	*P. juruazensis*	Pota_ju_3	CORBIDI 15550	KU902156	KU902238		KU902313	KU902075	Torres-Carvajal et al. 2016
Potamites	*P. juruazensis*	Pota_ju_4	CORBIDI 15579	KU902157	KU902239		KU902314	KU902076	Torres-Carvajal et al. 2016
Potamites	*P. juruazensis*	Pota_ju_5	CORBIDI 15479		KU902236		KU902311	KU902073	Torres-Carvajal et al. 2016
Potamites	*P. montanicola*	Pota_mo_1	CORBIDI 10791	KU902158	KU902240		KU902315	KU902077	Torres-Carvajal et al. 2016
Potamites	*P. strangulatus*	Pot_str_1	KU21677	AY507847	AY507866		AY507885		Doan et al. 2005
Potamites	*P. strangulatus*	Pota_st_1	QCAZ 6133	KU902159	KU902241		KU902316	KU902078	Torres-Carvajal et al. 2016
Potamites	*P. trachodus*	Pota_tr_2	CORBIDI 15489	KU902160	KU902242		KU902317	KU902079	Torres-Carvajal et al. 2016
Potamites	*P. trachodus*	Pota_tr_3	CORBIDI 15514	KU902161	KU902243		KU902318	KU902080	Torres-Carvajal et al. 2016
Potamites	*P. trachodus*	Pota_tr_4	CORBIDI 15515	KU902162	KU902244		KU902319	KU902081	Torres-Carvajal et al. 2016
Proctoporus	*P. bolivianus*	Pro_bol_1	UTA R-52944	AY968826	AY968833		AY968814		Doan et al. 2005
Proctoporus	*P. bolivianus*	Pro_bol_10	MNCN43664	JX435938	JX435999			JX436038	Goicoechea et al. 2012
Proctoporus	*P. bolivianus*	Pro_bol_11	MNCN43678	JX435939			JX436067	JX436039	Goicoechea et al. 2012
Proctoporus	*P. bolivianus*	Pro_bol_12	MNCN 8989	JX435940	JX435994		JX436071	JX436040	Goicoechea et al. 2012
Proctoporus	*P. bolivianus*	Pro_bol_13	MNCN 8990	JX435941	JX435995		JX436070	JX436041	Goicoechea et al. 2012
Proctoporus	*P. bolivianus*	Pro_bol_14	MNCN8991	JX435942	JX435996		JX436068	JX436042	Goicoechea et al. 2012
Proctoporus	*P. bolivianus*	Pro_bol_15	MNCN43679	JX435943	JX435997		JX436069	JX436043	Goicoechea et al. 2012
Proctoporus	*P. bolivianus*	Pro_bol_2	UTA R-52946	AY968822	AY968831		AY968811		Doan et al. 2005
Proctoporus	*P. bolivianus*	Pro_bol_3	MNCN43660	JX435931	JX435989		JX436061		Goicoechea et al. 2012
Proctoporus	*P. bolivianus*	Pro_bol_4	MHNC5333	JX435932	JX435990		JX436064	JX436033	Goicoechea et al. 2012
Proctoporus	*P. bolivianus*	Pro_bol_5	MHNC5334	JX435933	JX435991		JX436062		Goicoechea et al. 2012
Proctoporus	*P. bolivianus*	Pro_bol_6	MHNC5348	JX435935	JX435992			JX436034	Goicoechea et al. 2012
Proctoporus	*P. bolivianus*	Pro_bol_7	MNCN43662	JX435934	JX435993		JX436063	JX436035	Goicoechea et al. 2012
Proctoporus	*P. bolivianus*	Pro_bol_8	MHNC5357	JX435936	JX435998		JX436065	JX436036	Goicoechea et al. 2012
Proctoporus	*P. bolivianus*	Pro_bol_9	MNCN43663	JX435937	JX436000		JX436066	JX436037	Goicoechea et al. 2012
Proctoporus	*P.* Ca1	Pro_bol_16	MHNC5346	JX435944			JX436099		Goicoechea et al. 2012
Proctoporus	*P.* Ca1	Pro_bol_17	MHNC5322	JX435945	JX435988			JX436045	Goicoechea et al. 2012
Proctoporus	*P.* Ca1	Pro_bol_18	UTA R-52945	AY968825	AY968832		AY968813		Doan et al. 2005
Proctoporus	*P.* Ca2	Pro_bol_19	AMNH R-150695	AY507851	AY968828		AY968812		Doan et al. 2005
Proctoporus	*P. carabaya*	Pro_bol_36	MHNC5428	JX435912	JX435979		JX436083	JX436016	Goicoechea et al. 2012<br>Torres-Carvajal et al. 2016

Genus	Species	Sample code	Voucher	GenBank Accession					Source
				12S	16S	cytb	ND4	c-mos	
Proctoporus	*P. carabaya*	Pro_bol_37	MNCN43675	JX435913	JX435980		JX436084	JX436017	Goicoechea et al. 2012<br>Torres-Carvajal et al. 2016
Proctoporus	*P. carabaya*	Pro_bol_38	MNCN43676	JX435914	JX435981		JX436085	JX436018	Goicoechea et al. 2012<br>Torres-Carvajal et al. 2016
Proctoporus	*P. carabaya*	Pro_bol_39	MHNC5429	JX435915	JX435982		JX436086	JX436019	Goicoechea et al. 2012<br>Torres-Carvajal et al. 2016
Proctoporus	*P. chasqui*	IWU133	MUSM 31172	MH579605	MH579641	MH579671		MH579697	This study
Proctoporus	*P. chasqui*	IWU24	MUSM 31108	MH579601	MH579637	MH579667		MH579693	This study
Proctoporus	*P. chasqui*	IWU25	MUSM 31109	MH579602	MH579638	MH579668		MH579694	This study
Proctoporus	*P. chasqui*	IWU50	MUSM 31123	MH579603	MH579639	MH579669		MH579695	This study
Proctoporus	*P. chasqui*	IWU82	MUSM 31142	MH579604	MH579640	MH579670		MH579696	This study
Proctoporus	*P. chasqui*	Pro_chas_1	MNCN6771	JX435887	JX435946		JX436051	JX436003	Goicoechea et al. 2012
Proctoporus	*P. chasqui*	Pro_chas_2	MNCN44407	JX435888	JX435947		JX436052	JX436004	Goicoechea et al. 2012
Proctoporus	*P. chasqui*	Pro_chas_3	MNCN44408	JX435889	JX435948		JX436053	JX436005	Goicoechea et al. 2012
Proctoporus	*P. chasqui*	Proc_ch_1	CORBIDI 8416	KU902166	KU902246		KU902323	KU902085	Torres-Carvajal et al. 2016
Proctoporus	*P. chasqui*	Proc_ch_2	CORBIDI 8431	KU902167	KU902247		KU902324	KU902086	Torres-Carvajal et al. 2016
Proctoporus	*P. chasqui*	Proc_ch_3	CORBIDI 8478	KU902168	KU902248		KU902325	KU902087	Torres-Carvajal et al. 2016
Proctoporus	*P. chasqui*	Pro_sp_1	MHNC6834	JX435890	JX435949		JX436054	JX436006	Goicoechea et al. 2012
Proctoporus	*P. guentheri*	Pro_gue_1	UTA R-51515	AY507849	AY507872		AY225185	AY507900	Goicoechea et al. 2012
Proctoporus	*P. guentheri*	Pro_gue_2	UTA R-51517	AY507854	AY507873		AY225169	AY507901	Goicoechea et al. 2012
Proctoporus	*P. iridescens*	Pro_bol_21	MHNC5359	JX435923	JX435958		JX436074	JX436009	Goicoechea et al. 2012
Proctoporus	*P. iridescens*	Pro_bol_22	MHNC6360	JX435924	JX435959		JX436075	JX436010	Goicoechea et al. 2012
Proctoporus	*P. iridescens*	Pro_bol_23	MHNC5361	JX435925	JX435960		JX436076		Goicoechea et al. 2012
Proctoporus	*P. iridescens*	Pro_bol_25	MHNC5417	JX435916	JX435963		JX436088	JX436015	Goicoechea et al. 2012
Proctoporus	*P. iridescens*	Pro_bol_26	MHNC5421	JX435917	JX435964		JX436087		Goicoechea et al. 2012
Proctoporus	*P. iridescens*	Pro_bol_27	MNCN43669	JX435918	JX435965		JX436090	JX436031	Goicoechea et al. 2012
Proctoporus	*P. iridescens*	Pro_bol_28	MNCN44222	JX435919	JX435985		JX436077		Goicoechea et al. 2012
Proctoporus	*P. iridescens*	Pro_bol_29	MNCN44223	JX435926	JX435986			JX436020	Goicoechea et al. 2012
Proctoporus	*P. iridescens*	Pro_bol_32	MHNC4600	JX435928	JX435974		JX436080	JX436025	Goicoechea et al. 2012
Proctoporus	*P. iridescens*	Pro_bol_33	MHNC4661	JX435929	JX435975		JX436081	JX436027	Goicoechea et al. 2012
Proctoporus	*P. iridescens*	Pro_bol_34	MHNC4629	JX435930	JX435976		JX436082		Goicoechea et al. 2012
Proctoporus	*P. iridescens*	Pro_bol_35	MHNC6005	JX435927	JX435966		JX436079	JX436049	Goicoechea et al. 2012
Proctoporus	*P. iridescens*	Pro_bol_20	MNCN43666	JX435922	JX435957		JX436072	JX436008	Goicoechea et al. 2012<br>Torres-Carvajal et al. 2016
Proctoporus	*P. iridescens*	Pro_bol_24	MNCN43668	JX435911	JX435962		JX436089	JX436014	Goicoechea et al. 2012<br>Torres-Carvajal et al. 2016

Genus	Species	Sample code	Voucher	GenBank Accession					Source
				12S	16S	cytb	ND4	c-mos	
Proctoporus	*P. iridescens*	Pro_bol_30	MNCN44224	JX435920	JX435987		JX436078	JX436021	Goicoechea et al. 2012<br>Torres-Carvajal et al. 2016
Proctoporus	*P. iridescens*	Pro_bol_31	MHNC5651	JX435921	JX435961		JX436073		Goicoechea et al. 2012<br>Torres-Carvajal et al. 2016
Proctoporus	*P. kiziriani*	Proc_ca_1	CORBIDI 14709	KU902163			KU902320	KU902082	Torres-Carvajal et al. 2016
Proctoporus	*P. kiziriani*	Proc_ca_2	CORBIDI 14710	KU902164	KU902245		KU902321	KU902083	Torres-Carvajal et al. 2016
Proctoporus	*P. kiziriani*	Proc_ca_3	CORBIDI 14711	KU902165			KU902322	KU902084	Torres-Carvajal et al. 2016
Proctoporus	*P. kiziriani*	Pro_bol_40	MNCN43670	JX435906	JX435967			JX436032	Goicoechea et al. 2012
Proctoporus	*P. kiziriani*	Pro_bol_41	MHNC5367	JX435907	JX435978		JX436091	JX436011	Goicoechea et al. 2012
Proctoporus	*P. kiziriani*	Pro_bol_42	MHNC43671	JX435910	JX435968			JX436012	Goicoechea et al. 2012
Proctoporus	*P. kiziriani*	Pro_bol_43	MHNC5370	JX435909	JX435970		JX436092		Goicoechea et al. 2012
Proctoporus	*P. kiziriani*	Pro_bol_44	MHNC5371	JX435908	JX435969		JX436093	JX436013	Goicoechea et al. 2012
Proctoporus	*P. kiziriani*	Pro_bol_45	MNCN44216	JX435900	JX435972		JX436096	JX436022	Goicoechea et al. 2012
Proctoporus	*P. kiziriani*	Pro_bol_46	MNCN44217	JX435905	JX435971		JX436094		Goicoechea et al. 2012
Proctoporus	*P. kiziriani*	Pro_bol_47	MNCN44218	JX435903	JX435973			JX436023	Goicoechea et al. 2012<br>Torres-Carvajal et al. 2016
Proctoporus	*P. kiziriani*	Pro_bol_48	MHNC4750	JX435901	JX435984		JX436098	JX436046	Goicoechea et al. 2012
Proctoporus	*P. kiziriani*	Pro_bol_49	MHNC4751	JX435904	JX435977		JX436097	JX436048	Goicoechea et al. 2012<br>Torres-Carvajal et al. 2016
Proctoporus	*P. kiziriani*	Pro_bol_50	MNCN4221	JX435902	JX435983		JX436095	JX436047	Goicoechea et al. 2012<br>Torres-Carvajal et al. 2016
Proctoporus	*P. lacertus*	Pro_bol_51	UTA R-51484	AY968820	AY968827				Doan et al. 2005<br>Goicoechea et al. 2012<br>Torres-Carvajal et al. 2016
Proctoporus	*P. lacertus*	Pro_bol_52	UTA R-51487	AY507850	AY507868		AY225180	AY507897	Goicoechea et al. 2012
Proctoporus	*P. lacertus*	Pro_bol_53	UTA R-51506	AY507851	AY507869		AY225175	AY507898	Goicoechea et al. 2012
Proctoporus	*P. laudahnae*	Proc_la_1	CORBIDI 15558	KU902171	KU902250		KU902328	KU902090	Torres-Carvajal et al. 2016
Proctoporus	*P. laudahnae*	Proc_la_2	CORBIDI 15743	KU902172	KU902251		KU902329	KU902091	Torres-Carvajal et al. 2016
Proctoporus	*P. oreades*	Proc_or_1	CORBIDI 7217	KU902173	KU902252		KU902330	KU902092	Torres-Carvajal et al. 2016
Proctoporus	*P. oreades*	Proc_or_2	CORBIDI 7218	KU902174	KU902253		KU902331	KU902093	Torres-Carvajal et al. 2016
Proctoporus	*P. oreades*	Proc_or_3	CORBIDI 7225	KU902175	KU902254		KU902332	KU902094	Torres-Carvajal et al. 2016
Proctoporus	*P. pachyurus*	Pro_pach_4	UTA R-52949	AY968824	AY968834		AY968816		Doan et al. 2005
Proctoporus	*P. pachyurus*	Pro_pach_5	MHNC TMD1203	AY968823	AY968829		AY968815		Doan et al. 2005
Proctoporus	*P. pachyurus*	Proc_pa_1	CORBIDI 11807	KU902176			KU902333	KU902095	Torres-Carvajal et al. 2016
Proctoporus	*P. pachyurus*	Proc_pa_2	CORBIDI 11810	KU902177			KU902334	KU902096	Torres-Carvajal et al. 2016
Proctoporus	*P. pachyurus*	Proc_pa_3	CORBIDI 11811	KU902178	KU902255		KU902335	KU902097	Torres-Carvajal et al. 2016
Proctoporus	*P. rahmi*	Pro_pach_1	MHNC11439	JX435893	JX435950		JX436056	JX436050	Goicoechea et al. 2012

Genus	Species	Sample code	Voucher	GenBank Accession					Source
				12S	16S	cytb	ND4	c-mos	
Proctoporus	*P. rahmi*	Proc_ra_1	CORBIDI 14705	KU902179	KU902256		KU902336	KU902098	Torres-Carvajal et al. 2016
Proctoporus	*P. rahmi*	Proc_ra_2	CORBIDI 14706	KU902180	KU902257		KU902337	KU902099	Torres-Carvajal et al. 2016
Proctoporus	*P. rahmi*	Proc_ra_3	CORBIDI 14707	KU902181	KU902258		KU902338	KU902100	Torres-Carvajal et al. 2016
Proctoporus	*P.* sp.	Pro_pach_2	MHNC4599	JX435891	JX435952		JX436055	JX436024	Goicoechea et al. 2012
Proctoporus	*P.* sp. 1	Proc_s1_1	CORBIDI 14624	KU902188	KU902263		KU902344	KU902107	Torres-Carvajal et al. 2016
Proctoporus	*P.* sp. 1	Proc_s1_2	CORBIDI 14625	KU902189	KU902264		KU902345	KU902108	Torres-Carvajal et al. 2016
Proctoporus	*P.* sp. 1	Proc_s1_3	CORBIDI 14626	KU902190	KU902265		KU902346	KU902109	Torres-Carvajal et al. 2016
Proctoporus	*P.* sp. 2	Proc_s2_1	CORBIDI 9636	KU902191	KU902266		KU902347	KU902110	Torres-Carvajal et al. 2016
Proctoporus	*P.* sp. 2	Proc_s2_2	CORBIDI 10753	KU902192	KU902267		KU902348	KU902111	Torres-Carvajal et al. 2016
Proctoporus	*P.* sp. 2	Proc_s2_3	CORBIDI 10754	KU902193	KU902268		KU902349	KU902112	Torres-Carvajal et al. 2016
Proctoporus	*P.* sp. 2	Proc_s2_4	CORBIDI 10755	KU902194	KU902269		KU902350	KU902113	Torres-Carvajal et al. 2016
Proctoporus	*P.* sp. 3	Proc_s3_1	CORBIDI 14692	KU902169			KU902326	KU902088	Torres-Carvajal et al. 2016
Proctoporus	*P.* sp. 3	Proc_s3_2	CORBIDI 14693	KU902170	KU902249		KU902327	KU902089	Torres-Carvajal et al. 2016
Proctoporus	*P.* sp. 4	IWU358	MUSM 32727	MH579606	MH579642	MH579672		MH579698	This study
Proctoporus	*P.* sp. 5	Pro_pach_3	MHNC4689	JX435892	JX435951		JX436057	JX436026	Goicoechea et al. 2012
Proctoporus	*P. spinalis*	IWU119	MUSM 31162	MH579607	MH579643				This study
Proctoporus	*P. spinalis*	IWU120	IWU 120	MH579608	MH579644			MH579699	This study
Proctoporus	*P. spinalis*	Proc_sp_1	CORBIDI 7234	KU902182	KU902259		KU902339	KU902101	Torres-Carvajal et al. 2016
Proctoporus	*P. spinalis*	Proc_sp_2	CORBIDI 7241	KU902183	KU902260		KU902340	KU902102	Torres-Carvajal et al. 2016
Proctoporus	*P. spinalis*	Proc_sp_3	CORBIDI 7246	KU902184	KU902261		KU902341	KU902103	Torres-Carvajal et al. 2016
Proctoporus	*P. spinalis*	Proc_sp_4	CORBIDI 11573	KU902185			KU902342	KU902104	Torres-Carvajal et al. 2016
Proctoporus	*P. spinalis*	Proc_sp_5	CORBIDI 11575	KU902186			KU902343	KU902105	Torres-Carvajal et al. 2016
Proctoporus	*P. sucullucu*	Pro_suc_1	MNCN44474	JX435894	JX435953			JX436028	Goicoechea et al. 2012
Proctoporus	*P. sucullucu*	Pro_suc_2	MNCN44475	JX435895	JX435954		JX436058	JX436029	Goicoechea et al. 2012
Proctoporus	*P. sucullucu*	Pro_suc_3	MNCN44476	JX435897	JX435955		JX436060	JX436030	Goicoechea et al. 2012
Proctoporus	*P. sucullucu*	Pro_suc_4	MNCN44478	JX435896	JX435956		JX436059	JX436044	Goicoechea et al. 2012
Proctoporus	*P. sucullucu*	Pro_suc_5	UTA R-52950		AY968830		AY968817		Doan et al. 2005
Proctoporus	*P. unsaacae*	Pro_uns_3	UTA R-51488	AY507859	AY507882		AY225186	AY507908	Doan et al. 2005
Proctoporus	*P. unsaacae*	Pro_uns_4	UTA R-51477	AY507860	AY507881		AY225170	AY507909	Doan et al. 2005
Proctoporus	*P. unsaacae*	Proc_un_1	CORBIDI 10562	KU902187	KU902262			KU902106	Torres-Carvajal et al. 2016
Proctoporus	*P. xestus*	Pro_xes_1	MNCN 6160	JX435898	JX436002		JX436101		Goicoechea et al. 2012
Proctoporus	*P. xestus*	Pro_xes_2	MNCN 2425	JX435899	JX436001		JX436100	JX436007	Goicoechea et al. 2012
Riama	*R.* "Cordillera Central"	Ria_CC1	A1	KY670708	KY681126			KY670670	Sanchez-Pacheco et al. 2017
Riama	*R.* "Cordillera Central"	Ria_CC2	A2	KY670709	KY681127			KY670671	Sanchez-Pacheco et al. 2017

Genus	Species	Sample code	Voucher	GenBank Accession					Source
				12S	16S	cytb	ND4	c-mos	
Riama	*R.* "Narino"	Ria_N1	SSP58	KY670711	KY681129		KY710849	KY670673	Sanchez-Pacheco et al. 2017
Riama	*R.* "Narino"	Ria_N2	SSP76	KY670712	KY681130		KY710850	KY670674	Sanchez-Pacheco et al. 2017
Riama	*R.* “Cordillera Occidental"	Ria_CO1	JJM2251	KY670710	KY681128		KY799162	KY670672	Sanchez-Pacheco et al. 2017
Riama	*R. anatoloros*	Ria_an_2	QCAZ9169	KY670702	KY681120		KY710843	KY670664	Sanchez-Pacheco et al. 2017
Riama	*R. anatoloros*	Ria_an_3	QCAZ9201	KY670703	KY681121		KY710844	KY670665	Sanchez-Pacheco et al. 2017
Riama	*R. anatoloros*	Ria_an_1	QCAZ 9203	KU902195	KU902270		KU902351	KU902114	Torres-Carvajal et al. 2016
Riama	*R. balneator*	Ria_ba_1	QCAZ 11099	KU902196	KU902271		KU902352	KU902115	Torres-Carvajal et al. 2016
Riama	*R. balneator*	Ria_ba_2	QCAZ11101	KY670704	KY681122		KY710845	KY670666	Sanchez-Pacheco et al. 2017
Riama	*R. cashcaensis*	Ria_cas_2	QCAZ10686	KJ948180	KJ948122		KJ948162	KJ948210	Aguirre-Penafiel et al. 2014
Riama	*R. cashcaensis*	Ria_cas_3	QCAZ 10754	KJ948181	KJ948126		KJ948163	KJ948219	Aguirre-Penafiel et al. 2014
Riama	*R. colomaromani*	Ria_col_1	KU217209	AY507853	AY507871		AY507888	AY507899	Doan et al. 2005
Riama	*R. colomaromani*	Ria_col_2	QCAZ8753	KY670705	KY681123		KY710846	KY670667	Sanchez-Pacheco et al. 2017
Riama	*R. columbiana*	Ria_columb_1	ICN11298	KY670706	KY681124		KY710847	KY670668	Sanchez-Pacheco et al. 2017
Riama	*R. columbiana*	Ria_columb_2	ICN11294	KY670707	KY681125		KY710848	KY670669	Sanchez-Pacheco et al. 2017
Riama	*R. labionis*	Ria_lab_2	QCAZ10412	KJ948172	KJ948121		KJ948148	KJ948207	Aguirre-Penafiel et al. 2014
Riama	*R. labionis*	Ria_lab_1	QCAZ 10411	KJ948171	KJ948120		KJ948147	KJ948218	Aguirre-Penafiel et al. 2014
Riama	*R. meleagris*	Ria_mel_1	QCAZ9841	KJ948185	KJ948130		KJ948165	KJ948211	Aguirre-Penafiel et al. 2014
Riama	*R. meleagris*	Ria_mel_2	QCAZ9842	KJ948191	KJ948131		KJ948166	KJ948198	Aguirre-Penafiel et al. 2014
Riama	*R. meleagris*	Ria_mel_3	QCAZ9845	KJ948184	KJ948132		KJ948167	KJ948197	Aguirre-Penafiel et al. 2014
Riama	*R. meleagris*	Ria_mel_4	QCAZ9846	KJ948183	KJ948133		KJ948168	KJ948212	Aguirre-Penafiel et al. 2014
Riama	*R. meleagris*	Ria_mel_5	QCAZ 9840	KJ948182	KJ948129		KJ948164	KJ948214	Aguirre-Penafiel et al. 2014
Riama	*R. orcesi*	Ria_orc_1	KU221772	AY507855	AY507874		AY507889		Doan et al. 2005
Riama	*R. orcesi*	Ria_orc_2	QCAZ 10569	KU902197	KU902272		KU902353	KU902116	Torres-Carvajal et al. 2016
Riama	*R. orcesi*	Ria_orc_3	QCAZ9035	KY670713	KY681131		KY710851	KY670675	Sanchez-Pacheco et al. 2017
Riama	*R. raneyi*	Ria_ran_1	QCAZ10090	KY670714	KY681132		KY710852		Sanchez-Pacheco et al. 2017
Riama	*R. raneyi*	Ria_ran_2	QCAZ9034	KY670715	KY681133		KY710853		Sanchez-Pacheco et al. 2017
Riama	*R. simotera*	Ria_sim_1	KU 217207	AY507856	AY507875		AY507890	AY507902	Aguirre-Penafiel et al. 2014
Riama	*R. simotera*	Ria_sim_2	QCAZ4120	KY670716	KY681134		KY710854	KY670676	Sanchez-Pacheco et al. 2017
Riama	*R. simotera*	Ria_sp_1	QCAZ 879	AY507861	AY507877			AY507904	Castoe et al. 2004
Riama	*R. stigmatoral*	Ria_stig_1	QCAZ7374	KJ948187	KJ948128		KJ948161	KJ948217	Aguirre-Penafiel et al. 2014
Riama	*R. stigmatoral*	Ria_stig_2	QCAZ9946	KJ948192	KJ948123		KJ948160	KJ948208	Aguirre-Penafiel et al. 2014
Riama	*R. stigmatoral*	Ria_stig_3	QCAZ 11412	KJ948189	KJ948124		KJ948158	KJ948209	Aguirre-Penafiel et al. 2014
Riama	*R. stigmatoral*	Ria_stig_4	QCAZ11415	KJ948190	KJ948127		KJ948159	KJ948215	Aguirre-Penafiel et al. 2014
Riama	*R. striata*	Ria_str_1	MAR333	KY670717	KY681135			KY670677	Sanchez-Pacheco et al. 2017

Genus	Species	Sample code	Voucher	GenBank Accession					Source
				12S	16S	cytb	ND4	c-mos	
Riama	*R. striata*	Ria_str_2	MAR933	KY670718	KY681136		KY710855	KY670678	Sanchez-Pacheco et al. 2017
Riama	*R. unicolor*	Ria_uni_1	QCAZ8405	KJ948173	KJ948139		KJ948149	KJ948201	Aguirre-Penafiel et al. 2014
Riama	*R. unicolor*	Ria_uni_10	KU 217211	AY507862	AY507880		AY507893	AY507907	Castoe et al. 2004
Riama	*R. unicolor*	Ria_uni_11	QCAZ9662	KY670719	KY681137		KY710856	KY670679	Sanchez-Pacheco et al. 2017
Riama	*R. unicolor*	Ria_uni_2	QCAZ8407	KJ948175	KJ948135		KJ948151	KJ948206	Aguirre-Penafiel et al. 2014
Riama	*R. unicolor*	Ria_uni_3	QCAZ8409	KJ948179	KJ948136		KJ948152	KJ948199	Aguirre-Penafiel et al. 2014
Riama	*R. unicolor*	Ria_uni_4	QCAZ8411	KJ948178	KJ948141		KJ948153	KJ948200	Aguirre-Penafiel et al. 2014
Riama	*R. unicolor*	Ria_uni_5	QCAZ8800	KJ948174	KJ948134		KJ948155	KJ948203	Aguirre-Penafiel et al. 2014
Riama	*R. unicolor*	Ria_uni_6	QCAZ9664	KJ948196	KJ948119		KJ948150	KJ948222	Aguirre-Penafiel et al. 2014
Riama	*R. unicolor*	Ria_uni_7	QCAZ9681	KJ948177	KJ948140		KJ948154	KJ948202	Aguirre-Penafiel et al. 2014
Riama	*R. unicolor*	Ria_uni_8	QCAZ 9682	KJ948188	KJ948138		KJ948157	KJ948205	Aguirre-Penafiel et al. 2014
Riama	*R. unicolor*	Ria_uni_9	QCAZ9684	KJ948176	KJ948137		KJ948156	KJ948204	Aguirre-Penafiel et al. 2014
Riama	*R. yumborum*	Ria_yum_1	QCAZ10822	KJ948186	KJ948125		KJ948169	KJ948213	Aguirre-Penafiel et al. 2014
Riama	*R. yumborum*	Ria_yum_2	QCAZ 10827	KJ948195	KJ948142		KJ948170	KJ948216	Aguirre-Penafiel et al. 2014
Selvasaura	*S. brava*	IWU339	MUSM 32718	MH579609	MH579645	MH579673		MH579700	This study
Selvasaura	*S. brava*	IWU340	NMP6V 75655	MH579610	MH579646	MH579674		MH579701	This study
Selvasaura	*S. brava*	IWU380	NMP6V 75653	MH579611	MH579647	MH579675		MH579702	This study
Selvasaura	*S. brava*	IWU381	MUSM 32738	MH579612	MH579648	MH579676		MH579703	This study
Selvasaura	*S. brava*	IWU382	NMP6V 75654	MH579613	MH579649	MH579677		MH579704	This study
Selvasaura	*S.* sp.	Cerc_s3_1	CORBIDI 15117	KU902203	KU902278		KU902359	KU902122	Torres-Carvajal et al. 2016
Selvasaura	*S.* sp.	Cerc_s3_2	CORBIDI 15118	KU902204	KU902279		KU902360	KU902123	Torres-Carvajal et al. 2016
Selvasaura	*S.* sp.	Cerc_s3_3	CORBIDI 15119	KU902205	KU902280		KU902361	KU902124	Torres-Carvajal et al. 2016
Selvasaura	*S.* sp.	Cerc_s3_4	QCAZ 12798	KU902206	KU902281		KU902362	KU902125	Torres-Carvajal et al. 2016
Selvasaura	*S.* sp.	Cerc_s3_5	QCAZ 12891	KU902207	KU902282		KU902363	KU902126	Torres-Carvajal et al. 2016
Unnamed clade 2		90	NMP6V 75090	MH579621	MH579656				This study
Unnamed clade 2		91	NMP6V 75091	MH579622	MH579657				This study
Unnamed clade 2		Cerc_s2_1	CORBIDI 8815	KU902200	KU902275		KU902356	KU902119	Torres-Carvajal et al. 2016
Unnamed clade 2		Cerc_s2_2	CORBIDI 13634	KU902201	KU902276		KU902357	KU902120	Torres-Carvajal et al. 2016
Unnamed clade 2		Cerc_s2_3	CORBIDI 13636	KU902202	KU902277		KU902358	KU902121	Torres-Carvajal et al. 2016
Unnamed clade 2		IWU114	MUSM 31160	MH579614		MH579679		MH579706	This study
Unnamed clade 2		IWU165	MUSM 31188	MH579615	MH579651	MH579680		MH579707	This study
Unnamed clade 2		IWU287	MUSM 32973	MH579616				MH579708	This study
Unnamed clade 2		IWU288	NMP6V 75084	MH579617	MH579652	MH579681		MH579709	This study
Unnamed clade 2		IWU296	MUSM 31978	MH579618	MH579653	MH579682			This study

Genus	Species	Sample code	Voucher	GenBank Accession					Source
				12S	16S	cytb	ND4	c-mos	
Unnamed clade 2		IWU320	MUSM 31991	MH579619	MH579654	MH579683		MH579710	This study
Unnamed clade 2		IWU325	MUSM 31994	MH579620	MH579655	MH579684		MH579711	This study
Unnamed clade 2		IWU57	MUSM 31127		MH579650	MH579678		MH579705	This study
Unnamed clade 4		EL409	MUSM 27610	MH579623					This study
Unnamed clade 4		ML1352	MUSM 25345	MH579624	MH579658	MH579685			This study
				**outgroup**					
Alopoglossus	*A. viridiceps*	Alop_vi_1	QCAZ 10670	KP874789	KP874841		KJ705316	KP874899	Torres-Carvajal et al. 2014
Bachia	*B. barbouri*	Bach_bar_1		DQ383205	DQ383216			DQ383193	Kohlsdorf and Wagner 2006
Bachia	*B. bicolor*	Bach_bic_1			DQ383217			DQ383194	Kohlsdorf and Wagner 2006
Bachia	*B. bresslaui*	Bach_bre_1	MRT 916883		AF420755		AF420876	AF420860	Pellegrino et al. 2001
Bachia	*B. dorbignyi*	Bach_dor_1	MRT 977273	AF420688	AF420754		AF420892		Pellegrino et al. 2001<br>Teixeira et al. 2013
Bachia	*B. flavescens*	Bach_fla_1	LSUMZ H12977	AF420705	AF420753		AF420869	AF420859	Pellegrino et al. 2001<br>Teixeira et al. 2013
Bachia	*B. heteropa*	Bach_het_1		DQ383202	DQ383222			DQ383199	Kohlsdorf and Wagner 2006
Bachia	*B. heteropa*	Bach_het_2		DQ383210	DQ383213			DQ383190	Kohlsdorf and Wagner 2006
Bachia	*B. huallagana*	Bach_hua_1		DQ383212	DQ383224			DQ383201	Kohlsdorf and Wagner 2006
Bachia	*B. intermedia*	Bach_int_1		DQ383204	DQ383215			DQ383192	Kohlsdorf and Wagner 2006
Bachia	*B. monodactylus*	Bach_mon_2		DQ383208	DQ383220			DQ383191	Kohlsdorf and Wagner 2006
Bachia	*B. m. parkerii*	Bach_mon_1		DQ383203	DQ383214			DQ383197	Kohlsdorf and Wagner 2006
Bachia	*B. panoplia*	Bach_pan_1		DQ383207	DQ383219			DQ383196	Kohlsdorf and Wagner 2006
Bachia	*B. peruana*	Bach_per_1		DQ383206	DQ383218			DQ383195	Kohlsdorf and Wagner 2006
Bachia	*B. scaea*	Bach_sca_1	H1119	KC597259	KC597262			KC597265	Teixeira et al. 2013
Bachia	*B. scaea*	Bach_sca_2	H1978	KC597258	KC597261			KC597264	Teixeira et al. 2013
Bachia	*B. scaea*	Bach_sca_3	H510	KC597257	KC597260			KC597263	Teixeira et al. 2013
Bachia	*B. scolecoides*	Bach_sco_1		DQ383211	DQ383223			DQ383200	Kohlsdorf and Wagner 2006
Bachia	*B. trisanale*	Bach_tri_1		DQ383209	DQ383221			DQ383198	Kohlsdorf and Wagner 2006
Ecpleopus	*E. gaudichaudii*	Ecpl_ga_1	LG 1356	AF420660	AF420738		AF420901	AF420855	Pellegrino et al. 2001
Gymnophthalmus	*G. leucomystax*	Gymn_le_1	MRT 946613	AF420675	AF420715		AF420906	AF420824	Pellegrino et al. 2001
Rhachisaurus	*R. brachylepis*	Rhac_br_1	MRT 887336	AF420665	AF420737		AF420877	AF420853	Pellegrino et al. 2001
Riolama	*R. inopinata*	Rio_ino_1	IRSNB 2680		KP283384		KP283395	KP283388	Kok 2015
Riolama	*R. leucosticta*	Rio_leu_1	IRSNB18152		JQ742254		KP283398	KP283391	Kok 2015
Riolama	*R. leucosticta*	Rio_leu_2	IRSNB18153		JQ742255		KP283397	KP283390	Kok 2015
Riolama	*R. leucosticta*	Rio_leu_3	VUB 3767		JQ742256		KP283396	KP283389	Kok 2015

References to Table S1.

Aguirre-Peñafiel, V., Torres-Carvajal, O., Nunes, P. M. S., Peck, M. R., & Maddock, S. T. (2014). A new species of *Riama* Gray, 1858 (Squamata: Gymnophthalmidae) from the Tropical Andes. *Zootaxa, 3866*(2), 246-260.

Castoe, T. A., Doan, T. M., & Parkinson, C. L. (2004). Data partitions and complex models in Bayesian analysis: the phylogeny of gymnophthalmid lizards. *Systematic Biology, 53*, 448–469.

Doan, T. M., Castoe, T. A., & Arriaga, W. A. (2005). Phylogenetic relationships of the genus *Proctoporus* sensu stricto (Squamata: Gymnophthalmidae), with a new species from Puno, southeastern Peru. *Herpetologica, 61*(3), 325–336.

Fu, J. (2000). Toward the phylogeny of the family Lacertidae – Why 4708 base pairs of mDNA sequences cannot draw the picture. *Biological Journal of the Linnean Society, 71*, 203-217.

Goicoechea, N., Padial, J. M., Chaparro, J. C., Castroviejo-Fisher, S., & De la Riva, I. (2012). Molecular phylogenetics, species diversity, and biogeography of the Andean lizards of the genus *Proctoporus* (Squamata: Gymnophthalmidae). *Molecular Phylogenetics and Evolution, 65*(3), 953-964.

Kohlsdorf, T., & Wagner, G. P. (2006). Evidence for the reversibility of digit loss: a phylogenetic study of limb evolution in *Bachia* (Gymnophthalmidae: Squamata). *Evolution, 60*(9), 1896-1912.

Kok, P. J. (2015). A new species of the Pantepui endemic genus *Riolama* (Squamata: Gymnophthalmidae) from the summit of Murisipán-tepui, with the erection of a new gymnophthalmid subfamily. *Zoological Journal of the Linnean Society, 174*(3), 500-518.

Kok, P. J., MacCulloch, R. D., Means, D. B., Roelants, K., Van Bocxlaer, I., & Bossuyt, F. (2012). Low genetic diversity in tepui summit vertebrates. *Current Biology, 22*(15), R589-R590.

Pellegrino, K., Rodrigues, M. T., Yonenaga‐Yassuda, Y., & Sites, J. W. (2001). A molecular perspective on the evolution of microteiid lizards (Squamata, Gymnophthalmidae), and a new classification for the family. *Biological Journal of the Linnean Society, 74*(3), 315-338.

Sánchez‐Pacheco, S. J., Torres‐Carvajal, O., Aguirre‐Peñafiel, V., Nunes, P. M., Verrastro, L., Rivas, G. A., et al. (2017). Phylogeny of *Riama* (Squamata: Gymnophthalmidae), impact of phenotypic evidence on molecular datasets, and the origin of the Sierra Nevada de Santa Marta endemic fauna. *Cladistics, 2017*, 1-32.

Sturaro, M. J., Avila-Pires, T. C., & Rodrigues, M. T. (2017). Molecular phylogenetic diversity in the widespread lizard *Cercosaura ocellata* (Reptilia: Gymnophthalmidae) in South America. *Systematics and biodiversity, 15*(6), 532-540.

Teixeira, M. J., Dal Vechio, F., Nunes, P. M. S., Mollo Neto, A., Lobo, L. M., Storti, L. F., et al. (2013). A new species of *Bachia* Gray, 1845 (Squamata: Gymnophthalmidae) from the western Brazilian Amazonia. *Zootaxa, 3636*(3), 401-420.

Torres-Carvajal, O., Lobos, S. E., Venegas, P. J., Chávez, G., Aguirre-Peñafiel, V., Zurita, D., et al. (2016). Phylogeny and biogeography of the most diverse clade of South American gymnophthalmid lizards (Squamata, Gymnophthalmidae, Cercosaurinae). *Molecular Phylogenetics and Evolution, 99*, 63-75.

Torres-Carvajal, O., & Mafla-Endara, P. (2013). Evolutionary history of Andean *Pholidobolus* and *Macropholidus* (Squamata: Gymnophthalmidae) lizards. *Molecular Phylogenetics and Evolution, 68*(2), 212-217.

Torres-Carvajal, O., Venegas, P. J., Lobos, S. E., Mafla-Endara, P., & Sales Nunes, P. M. (2014). A new species of *Pholidobolus* (Squamata: Gymnophthalmidae) from the Andes of southern Ecuador. *Amphibian & Reptile Conservation, 8*(1), 76-88.
